# NVS-ZP7-4 inhibits hepatocellular carcinoma tumorigenesis and promotes apoptosis via PI3K/AKT signaling

**DOI:** 10.1038/s41598-023-38596-7

**Published:** 2023-07-21

**Authors:** Qing Tong, Dong Yan, Yan Cao, Xiaogang Dong, Yimamumaimaitijiang Abula, Huan Yang, Panpan Kong, Mingyu Yi

**Affiliations:** 1 Department of Hepatopancreatobiliary Surgery, Affiliated Cancer Hospital of Xinjiang Medical University, Urumqi, Xinjiang China; 2 Department of Anesthesiology, The Third Xiangya Hospital of Central South University, No. 138, Tongzipo Road, Changsha City, 410000 Hunan China

**Keywords:** Drug development, Liver cancer

## Abstract

NVS-ZP7-4 was identified as a novel chemical reagent targeting the zinc input protein ZIP7, which accounts for the zinc surge from the apparatus to the cytoplasm. Since zinc dysregulation is related to multiple diseases, in this study, we aimed to identify the anti-tumor effects of NVS-ZP7-4 and explore the molecular mechanisms of NVS-ZP7-4 in hepatocellular carcinoma (HCC) progression. We found that NVS-ZP7-4 inhibited cell viability, caused cell cycle arrest, induced apoptosis, and inhibited the proliferation, migration, and invasion of HCCLM3 and Huh7 cells. We further investigated the inhibited activation of the phosphatidylinositol 3-kinase (PI3K)/Akt pathway was involved in the antitumor effect of NVS-ZP7-4 in HCC. Furthermore, NVS-ZP7-4 inhibited HCC tumor growth in vivo. The present study demonstrated that NVS-ZP7-4 is a promising therapeutic target for HCC by regulating PI3K/AKT signaling.

## Introduction

ZIP7 is a zinc input protein anchored in the ER/Golgi apparatus and accounts for the zinc surge from the apparatus to cytoplasm. NVS-ZP7-4 has been identified as a novel chemical reagent that targets ZIP7, resulting in zinc accumulation in the ER^1–4^. Importantly, zinc is known as a co-effector of more than 300 enzymes and plays a vital role in the regulation of cellular function. Dysregulation of zinc is associated with multiple diseases, including immunodeficiency and tumors^5,6^. Recent studies have also shown aberrant higher expression and activation of ZIP7 in some malignant tumors, including tamoxifen-resistant breast cancer, esophageal cancer, lung cancer, prostate cancer, gastric cancer, and hepatocellular carcinoma (HCC)^7–9^. Thus, NVS-ZP7-4 might improve treatment efficiency by blocking ZIP7 and arresting zinc surge. However, studies of the therapeutic efficiency and underlying mechanisms of NVS-ZP7-4 in tumors are rare.

HCC remains a significant health challenge worldwide, and it is crucial to develop effective treatment strategies for HCC^10,11^. Dysregulated apoptosis is a fundamental driver of HCC development. For example, mice with nuclear receptor TAK1 and receptor-interacting serine/threonine-protein kinase 3 ablation in parenchymal liver cells developed passivity to apoptosis, which was proven to be an essential transversion towards malignancy^12,13^. Thus, targeting apoptosis is an alternative and feasible pharmacological strategy for the treatment of HCC^14–16^. Sorafenib-induced apoptosis has been identified in multiple tumors and has therapeutic functions in HCC^17,18^. Importantly, a previous study confirmed that ZIP7 gene knockout significantly increased cellular apoptosis, as in the case of colorectal cancer cells and cervical cancer^19,20^. Therefore, it is essential to explore the efficiency and molecular mechanisms of the ZIP7 inhibitor NVS-ZP7-4 in HCC growth and progression to identify novel therapies.

The phosphatidylinositol 3-kinase (PI3K)/AKT signaling pathway is genetically altered and activated in human cancers, and it participates in accelerated cancer metabolism, proliferation, and survival^21–26^. Researchers have shown that activation of AKT is critical for HCC development in mouse models^27,28^. Of note, zinc has been identified as an essential second messenger in cellular biofunction^29^, and cytoplasmic zinc accumulation inhibits phosphatases, prevents dephosphorylation of tyrosine kinase receptors^29,30^, and results in activation of the PI3K pathway^31^. Based on this knowledge, inhibition of the zinc surge to the cytoplasm by NVS-ZP7-4 might provide a novel strategy for treatment via targeting PI3K/AKT signaling in HCC.

To the best of our knowledge, the treatment efficiency and underlying mechanisms of NVS-ZP7-4 have not been explored in the field of HCC; therefore, we aimed to reveal the anti-tumor functions of NVS-ZP7-4 and its underlying mechanisms in HCC treatment for the first time and provide a novel promising treatment for HCC.

## Methods

All methods were performed in accordance with relevant guidelines and regulations.

### Reagents and antibodies

Purified NVS-ZP7-4 (98.68%) (Cat: HY-114395) and PI3K activator 740 Y-P (HY-P0175) were purchased from MedChemExpress (NJ, USA). Antibodies for western blotting used in the present study were PARP1 (1:1,000, Abclone, China), BCL2 (1:2,000, Proteintech, China), caspase-3 (1:1,000, Abclone), cleaved caspase-3 (1:200, Sigma, USA), α-tubulin (1:10,000, Proteintech), BAX (1:10,000, Proteintech), AKT (1:1,000, Proteintech), p-AKT (1:1,000, Proteintech), cyclin D1 (1:1,000, Proteintech), and p-ZIP7 (1:1,000, Sigma), total ZIP7 (1:1,000, Proteintech).

### Cell culture

Huh7 (adult hepatocellular carcinoma), HCCLM3 (adult hepatocellular carcinoma) cells were used for this study and purchased from the Chinese Academy of Sciences (Shanghai, China). The cells were incubated with Dulbecco’s modified Eagle’s medium (DMEM; Gibco, USA), 10% fetal bovine serum (FBS; Gibco, USA), and 10% penicillin–streptomycin solution at 37 °C, 5% CO_2_ and standard humidity. Mouse primary hepatocytes were isolated using BALB/c nude mouse liver according to a reported protocol^32^. Firstly, liver was perfused using Ca^2+^-and Mg^2+^-free Hanks’ balanced salt solution (HBSS, Sigma) through the portal vein. Secondly, liver was perfused with 0.1% collagenase I solution (Sigma) in HBSS containing Ca^2+^ and Mg^2+^. A few minutes letter, liver was excised and dispersed into cold HBSS. The cell suspension was generated and filtered through 70-μm pore size nylon cell strainer (Sigma). The cell supernatant was centrifuged and pellets were re-suspended in DMEM with 10% FBS. Cells were cultured for further experiments.

### Cell viability assay and cell proliferation

To determine the cell viability after NVS-ZP7-4 treatment, cell counting kit-8 (CCK-8) was used to measure the viability of Huh7, HCCLM3 cells and mouse primary hepatocytes after different treatments. Cells were seeded in 96-well plates (5 × 10^3^/well) and treated with NVS-ZP7-4 or DMSO for 24 h. CCK-8 solution was then added to each well (10 μL/100 μL) and incubated for 2 h at 37 °C. The absorbance was measured at a wavelength of 450 nm. Cell viability (%) = [Absorb(drug) − Absorb(blank)]/[Absorb(control) − Absorb(blank)].

For cell proliferation, cells were seeded 96-well plates (5 × 10^3^/well) and treated with different reagents. After 0 h, 24 h, 48 h, 72 h, cells were added with CCK-8 solution (10 μL/100 μL) and incubated for 2 h at 37 °C. The absorbance was measured at a wavelength of 450 nm.

### Apoptosis assays

The cells were treated with NVS-ZP7-4 or DMSO for 24 h before apoptosis detection. Cell suspensions were collected and stained with annexin V and propidium iodide (PI) in the dark for 15 min, and the cells were then analyzed with a flow cytometer.

### Cell cycle staining assay

Cells were seeded in 6-well plates and treated with various concentrations of NVS-ZP7-4 or DMSO for 24 h. Cell suspensions were collected, stained with PI, and analyzed using cell flow cytometry.

### Colony formation assay

Cells were seeded at 1,000 cells/well into six-well plates for the colony formation assay. After approximately 2 weeks of culture, the cells were fixed with 4% paraformaldehyde and stained with 0.1% crystal violet. Visible colonies (diameter > 0.1 mm) were photographed and counted.

### EdU assay

A 5-ethynyl-2′-deoxyuridine (EdU) assay (RIB-BIO) was conducted according to the manufacturer’s instructions. Briefly, cells were inoculated in a 96-well plate, incubated with 50 nM EdU-A reagent for 2 h, and then fixed and dyed using Apollo solution and Hoechst3342 solution. A fluorescence microscope AXIO Vert A1 (Carl Zeiss AG) was used for the photography.

### Migration and invasion assays

Only for transwell invasion assays, the upper chambers were coated with Matrigel film (Corning, USA) at 37 °C overnight before use. Both for transwell migration and invasion assays, cells were pretreated with NVS-ZP7-4 or DMSO for 24 h and resuspended in DMEM. Suspensions (200 μL) were re-seeded into the upper chamber, and 750 μL of DMEM with 10% FBS was added to each well. The cells were fixed with 4% paraformaldehyde after 48 h. Cells crossing the membrane were recorded using an EVOS XL Core Instrument (AMEX1,000, Thermo Fisher Scientific, USA) at 100 × and 200 × magnifications.

### Western blot analysis

Cells were inoculated in 6-well plates and treated with different reagents for 24 h. Then, the cells were collected and lysed for 30 min with RIPA buffer (Beyotime, Shanghai, China). The supernatant was harvested after centrifugation at 1.5 × 10^4^ rpm for 20 min. The protein concentration was detected using a BCA kit (Beyotime) and boiled with loading buffer (Beyotime) for 5 min. SDS-PAGE gels (12% or 10%) were used for protein electrophoresis, and the proteins were transferred to PVDF membranes (Millipore, MA, USA). Membranes were blocked and incubated with antibodies, and Super Signal West Atto (Thermo Scientific, MA, USA) were used for signal detection.

### In vivo tumor model

Five-week-old male, BALB/c nude mice (SJA Laboratory Animal Co. Ltd., Hunan, China) were raised under standard conditions of temperature, light, and free access to water and food. Mice were subcutaneously injected with 100 μL of Huh7 cells (2 × 10^6^ cells) to establish a subcutaneous HCC mouse model. The mice were randomly divided into two groups (n = 5): the control group received intraperitoneal injections of DMSO (0.1% in PBS, 2 mL/kg) three times a week, and the NVS-ZP7-4 group received NVS-ZP7-4 (1 mg/kg) three times a week. The mice were anesthetized with pentobarbital and sacrificed 2 weeks later. Tumor and body weights were recorded, and tumor tissues were collected for further immunohistochemical analysis. The study was approved by the Institutional Research Ethics Committees of Affiliated Tumor Hospital of Xinjiang Medical University (K-2021024). Mice were sacrificed by cervical dislocation. The study was reported in accordance with ARRIVE guidelines.

### Immunohistochemistry

Tumor tissues from HCC mice treated with NVS-ZP7-4 or DMSO were collected to detect the expression of AKT (1:200), p-AKT (1:200), proliferating cell nuclear antigen (PCNA) (1:500), and cleaved caspase-3 (1:20). Paraffin sections were dewaxed and boiled in a citrate solution for antigen repair. Then, 0.3% hydrogen peroxide was added, and the antibodies were incubated overnight. Sections were then incubated with secondary antibodies, stained with DAB staining kits (ZSGB-BIO, Beijing, China), stained with hematoxylin, and finally sealed with neutral balsam (Proteintech, China).

### Hematoxylin and eosin (H&E) staining

Paraffin sections were dewaxed, stained successively with hematoxylin and 0.5% eosin solution, dehydrated, and sealed with neutral balsam.

### Statistical analysis

For in vitro and in vivo examinations, data are presented as the mean ± standard deviation, with at least three repeats. ANOVA was used to measure the differences among groups, with a significant *p* value of < 0.05. All analyses were performed using GraphPad Prism 8 (GraphPad Software, San Diego, CA, USA).

### Ethical approval

The study was approved by the Institutional Research Ethics Committees of Affiliated Tumor Hospital of Xinjiang Medical University (K-2021024).

## Results

### Effects of NVS-ZP7-4 on the growth of HCC cells

**Figure 1:**
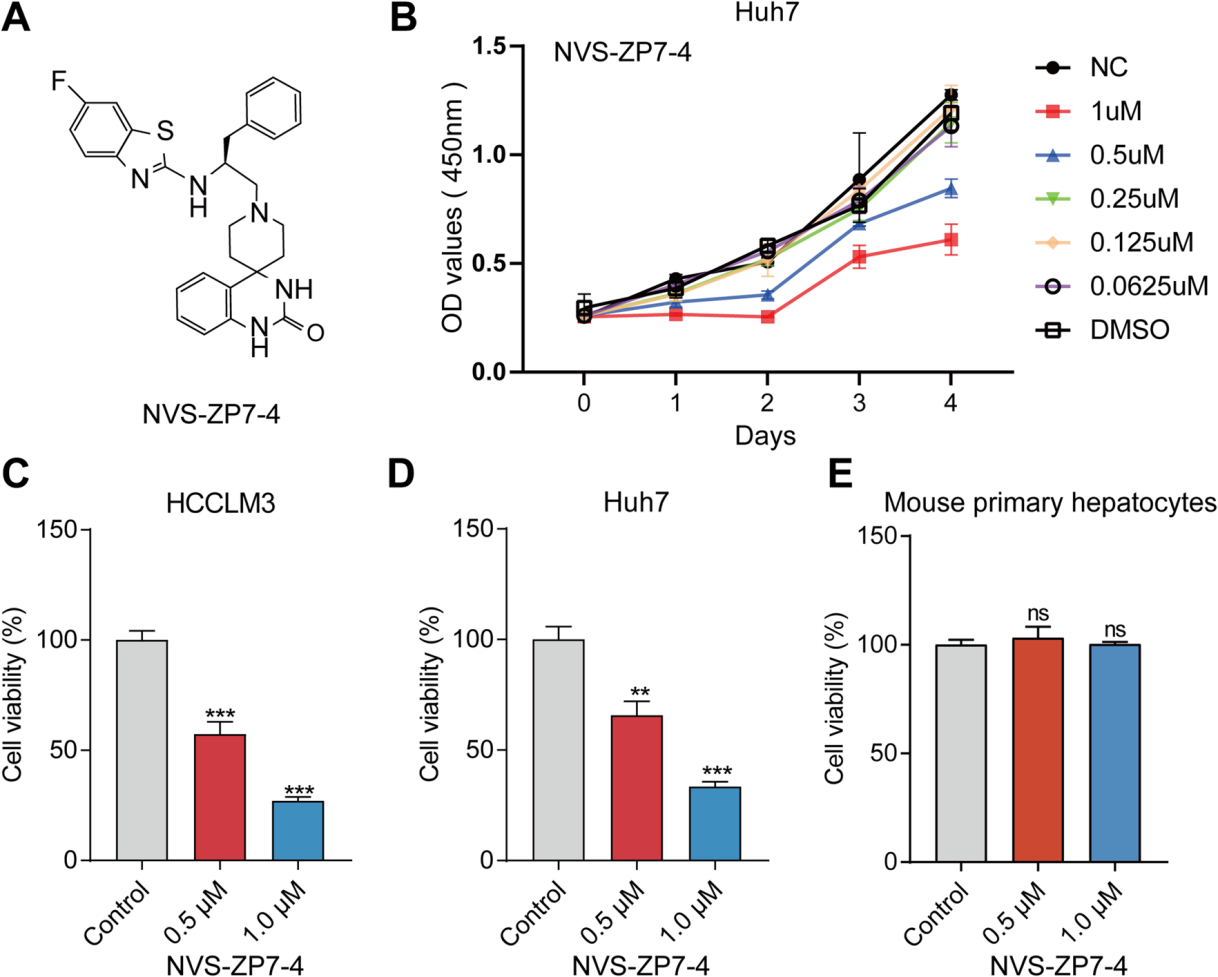
The effect of NVS-ZP7-4 on growth-inhibiting activity in HCCLM3, Huh7 cells and mouse primary hepatocytes. (**A**) The chemical structure of NVS-ZP7-4. Huh7 cells (**B**) were treated with NVS-ZP7-4 (0, 0.0625, 0.125, 0.25, 0.5, 1 μM), OD value was assayed by CCK-8. HCCLM3 (**C**) Huh7 cells (**D**) and mouse primary hepatocytes (**E**) were treated with NVS-ZP7-4 (0, 0.5, 1 μM) for 24 h. ns = no significance, **p < 0.01, ***p < 0.001 versus Control.

**Figure 2:**
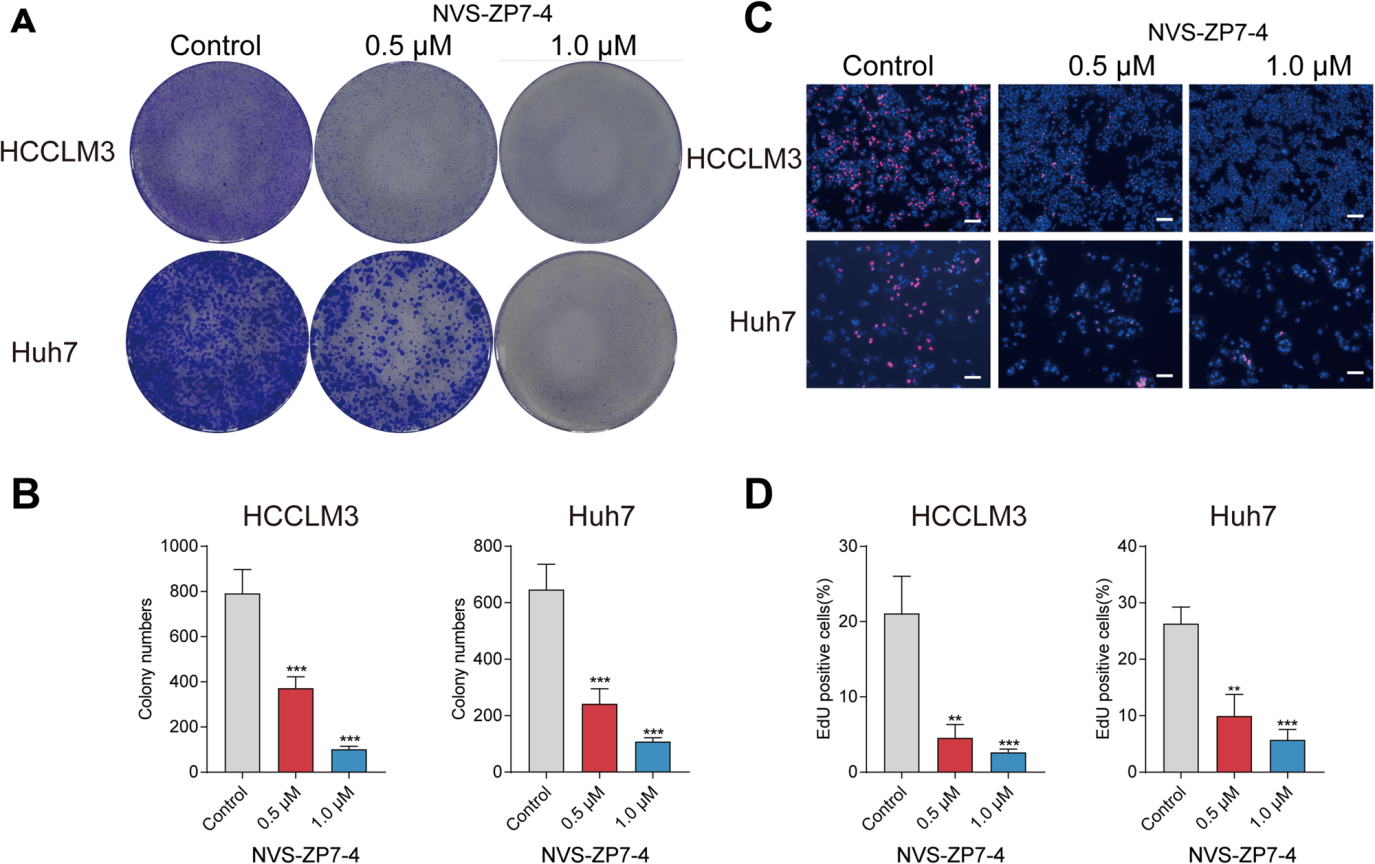
The effect of NVS-ZP7-4 on HCC cell proliferation. Representative cell colony images (**A**) and quantitation (**B**) of HCCLM3 and Huh7 cells. Representative EdU images (**C**) and quantitation (**D**) of HCCLM3 and Huh7 cells. HCCLM3 and Huh7 cells were treated with NVS-ZP7-4 (0, 0.5, 1 μM) for 24 h. **p < 0.01; ***p < 0.001 versus Control.

Huh7 cells were treated with NVS-ZP7-4 (chemical structure shown in Fig. 1A) of different concentrations (0, 0.0625, 0.125, 0.25, 0.5, 1 μM). With concentration at 0.5 and 1 μM, NVS-ZP7-4 showed best effects to inhibit the proliferation of Huh7 cells on cellular proliferation (Fig. 1B). HCCLM3, Huh7 cells and mouse primary hepatocytes were used to detect the effect of NVS-ZP7-4 on cell viability. The results for the cancer cell lines are shown in Fig. 1. Figure 1C and D shows that HCCLM3 and Huh7 cells displayed decreased cell viability after NVS-ZP7-4 treatment in a dose-dependent manner. Interestingly, cytotoxicity analysis showed that NVS-ZP7-4 was relatively less cytotoxic to mouse primary hepatocytes, suggesting the hypo-toxicity of NVS-ZP7-4 to normal liver cells (Fig. 1E). For further experiments, we used concentrations of 0.5 and 1 μM in HCCLM3 and Huh7 cells, and used 0.08% DMSO as control. The effect of NVS-ZP7-4 on the proliferation of HCCLM3 and Huh7 cells was detected using colony formation and EdU assays. The colony number and number of EdU-positive cells were significantly inhibited in a dose-dependent manner (Fig. 2), suggesting that NVS-ZP7-4 significantly inhibited HCC cell growth.

### NVS-ZP7-4 induced HCC cell apoptosis

**Figure 3:**
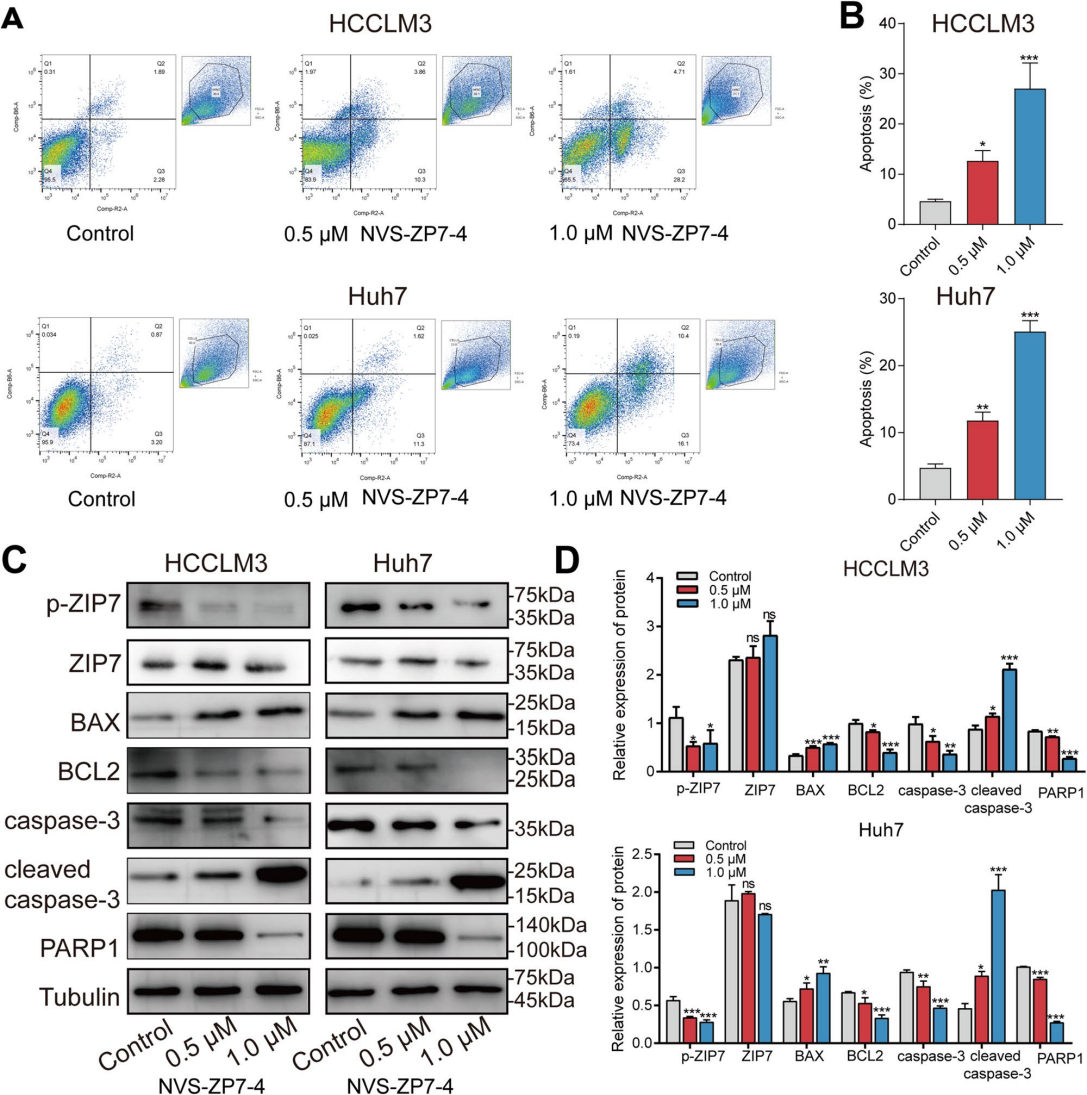
NVS-ZP7-4 induced apoptosis in HCC cells. Apoptosis was evaluated (**A**) and quantified (**B**) by flow cytometry. Core proteins in apoptosis were detected by western-blot. Representative bands (**C**) and quantitation (**D**) for ZIP7, p-ZIP7, BAX, BCL2, caspase-3, cleaved caspase-3 and PARP1 protein expression by western blot analysis. HCCLM3 and Huh7 cells were treated with NVS-ZP7-4 (0, 0.5, 1 μM) for 24 h. ns = no significance, *p < 0.05, **p < 0.01, ***p < 0.001 versus Control.

The PI3K/AKT pathway can prevent the programmed death of tumor cells and inhibit apoptosis, thus promoting the survival of tumor cells. Firstly, the apoptosis-positive cells were significantly induced in a dose-dependent manner as shown in Fig. 3A and B. Secondly, p-ZIP7 was significantly repressed after NVS-ZP7-4 treatment in HCCLM3 and Huh7 cells, but failed to regulate the expression of ZIP7 in cells. And then we examined the effect of NVS-ZP7-4 on cleavage of PARP1, which is part of the apoptotic cascade. Anti-apoptotic proteins within cells, such as BCL2, must be overwhelmed, and BAX should be activated to induce apoptosis. Consistently, as shown in Fig. 3C, D HCCLM3 and Huh7 cells treated with NVS-ZP7-4 had significantly decreased levels of PARP1, caspase-3, and BCL2 protein, as well as enhanced levels of cleaved caspase-3, and BAX protein expression. These data indicate that NVS-ZP7-4 activates caspase-dependent apoptosis in HCCLM3 and Huh7 cells. Besides, NVS-ZP7-4 treatment in vivo induced the expression of cleaved caspase-3 in a xenograft nude mouse model (Fig. 7D).

### NVS-ZP7-4 induced G0/G1 cell cycle arrest

**Figure 4:**
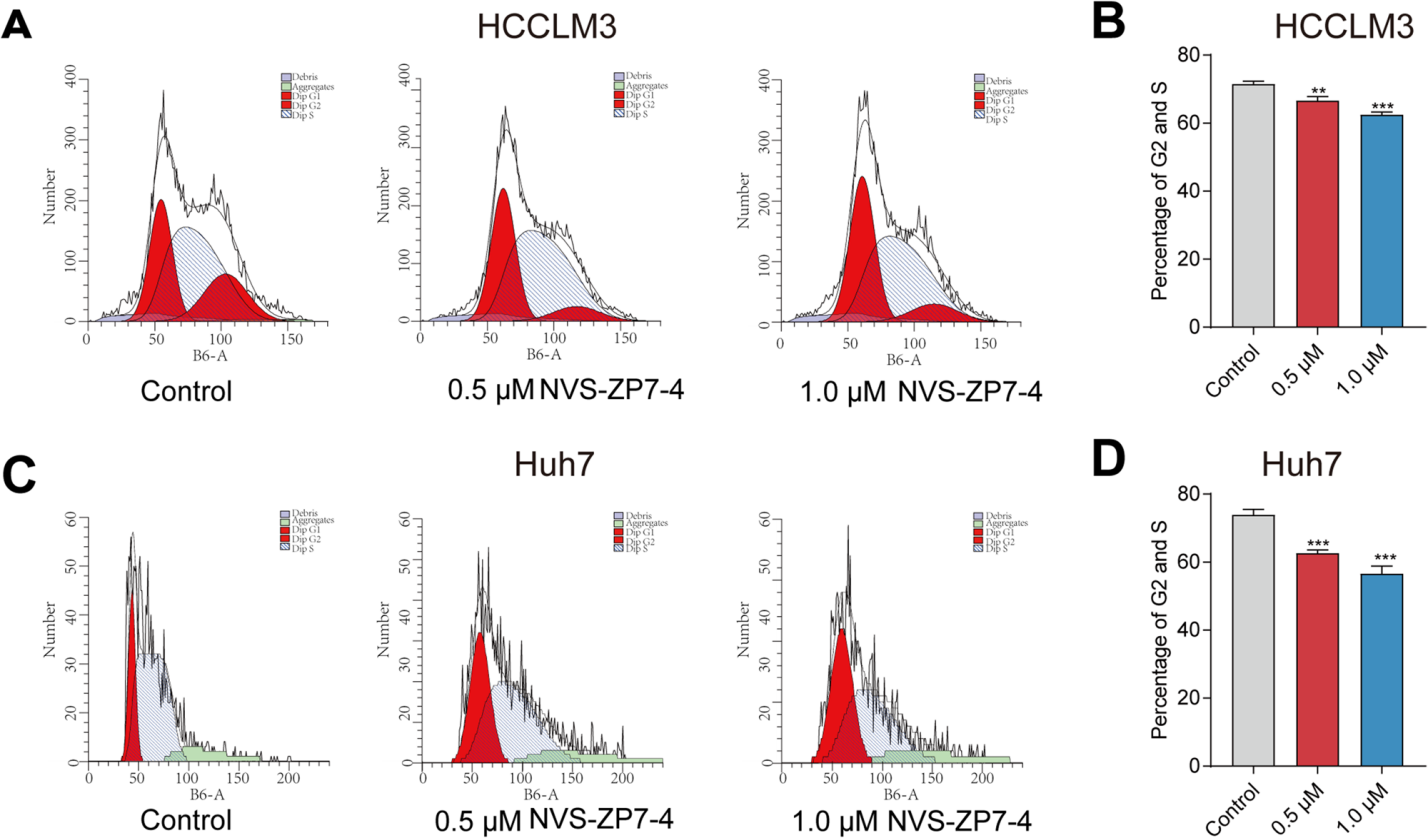
The effect of NVS-ZP7-4 on the cell cycle of HCC cells. (**A**) NVS-ZP7-4 treatment induced a dose dependent decrease in the proportion of cells in the G2 and S phase compared to the control. **p < 0.01, ***p < 0.001 versus Control.

To determine the mechanisms underlying inhibition of NVS-ZP7-4 on cell proliferation, flow cytometry was used to analyze DNA content for cell cycle analysis. As shown in Fig. 4, in the HCCLM3 and Huh7 cell lines, NVS-ZP7-4 induced increase in the proportion of cells in the G1 phase and a decrease in those in the G2, S phase. These results revealed that NVS-ZP7-4 induced cell cycle arrest in the G1 phase and had an anti-proliferative effect in the HCCLM3 and Huh7 cell lines.

### NVS-ZP7-4 inhibits HCC migration and invasion ability

**Figure 5:**
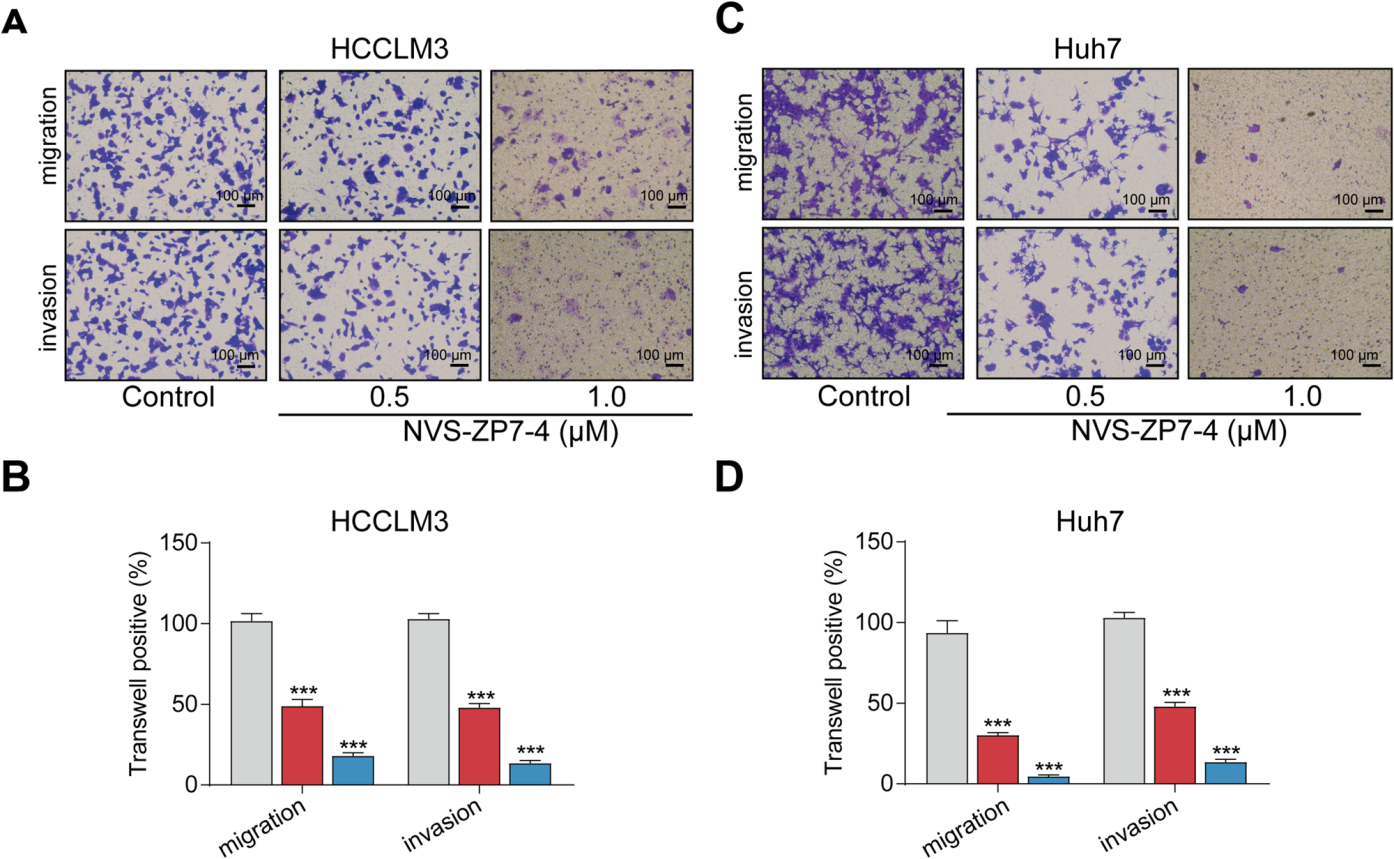
The effect of NVS-ZP7-4 on HCC cell migration and invasion. Representative images (**A**) and quantitation (**B**) of transwell migration and invasion assay. HCCLM3 and Huh7 cells were treated with NVS-ZP7-4 (0, 0.5, 1 μM) for 24 h. Scale bars: 100 μm. ***p < 0.001 versus Control.

PI3K/AKT signaling also participates in cancer-related metastasis; therefore, to determine the effect of NVS-ZP7-4 on HCC cell migration and invasion, we performed migration and invasion assays in HCCLM3 and Huh7 cells. As expected, NVS-ZP7-4 significantly inhibited the migration and invasion of HCCLM3 and Huh7 cells in a concentration-dependent manner (Fig. 5).

### NVS-ZP7-4 blocks PI3K/AKT signaling

**Figure 6:**
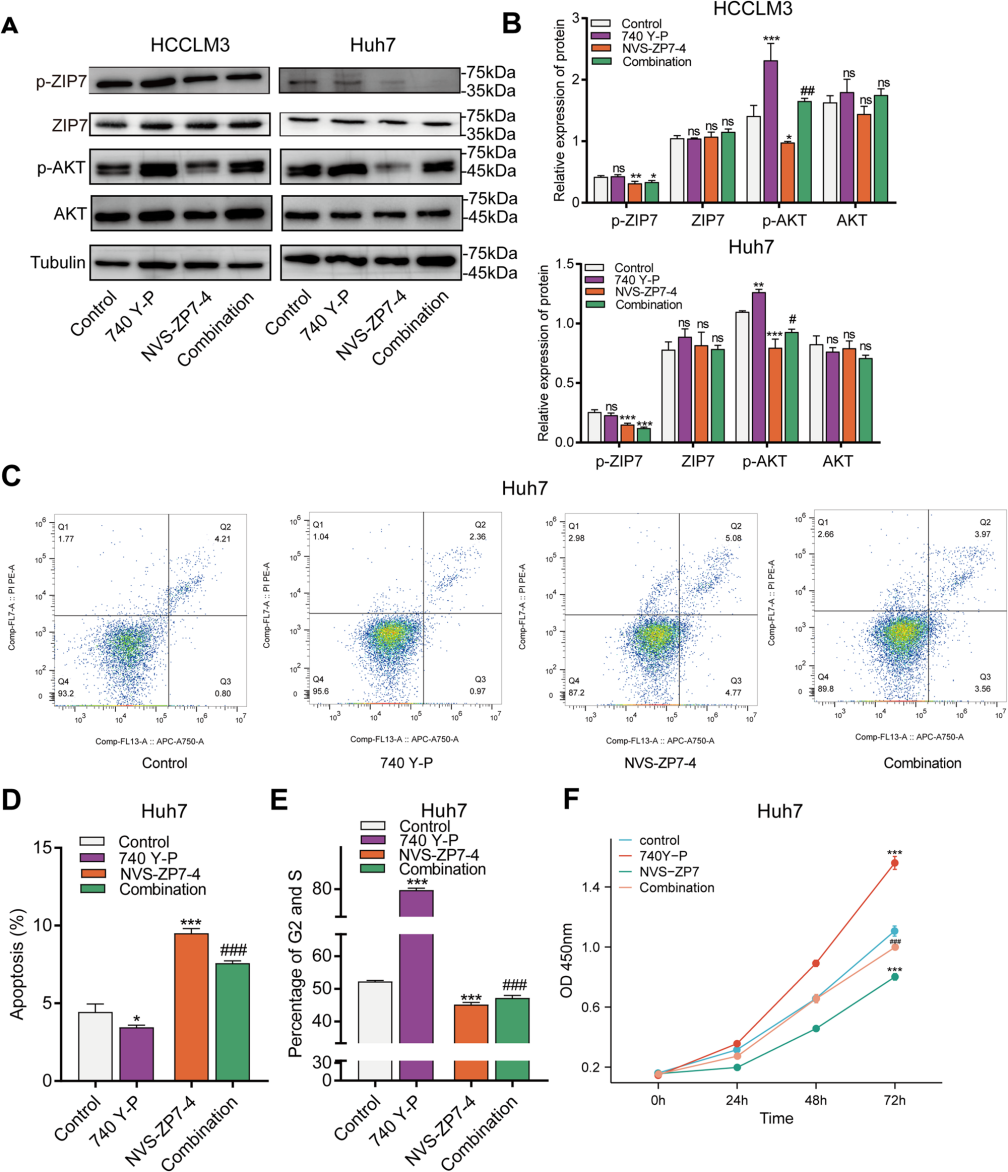
NVS-ZP7-4 inhibited PI3K/AKT pathway in HCC cells. HCCLM3 and Huh7 cells were treated with DMSO, 1 μM NVS-ZP7-4, PI3K activator 740 Y-P, or a combination of 1 μM NVS-ZP7-4 and 740 Y-P. (**A**,**B**) Western blotting analysis of p-ZIP7, ZIP7, and PI3K/AKT pathway targeted genes p-AKT, AKT in HCCLM3 and Huh7 cells after different treatment. (**C**,**D**) Apoptosis was evaluated (**C**) and quantified (**D**) by flow cytometry. (**E**) Cell cycle was evaluated and quantified by flow cytometry. (**F**) Cell proliferation was detected via CKK-8 at 0 h, 24 h, 48 h, 72 h. ns = no significance, *p < 0.05, **p < 0.01, ***p < 0.001 versus Control. #*p* < 0.05, ##*p* < 0.01; ###*p* < 0.001 versus the 1 μM NVS-ZP7-4.

**Figure 7:**
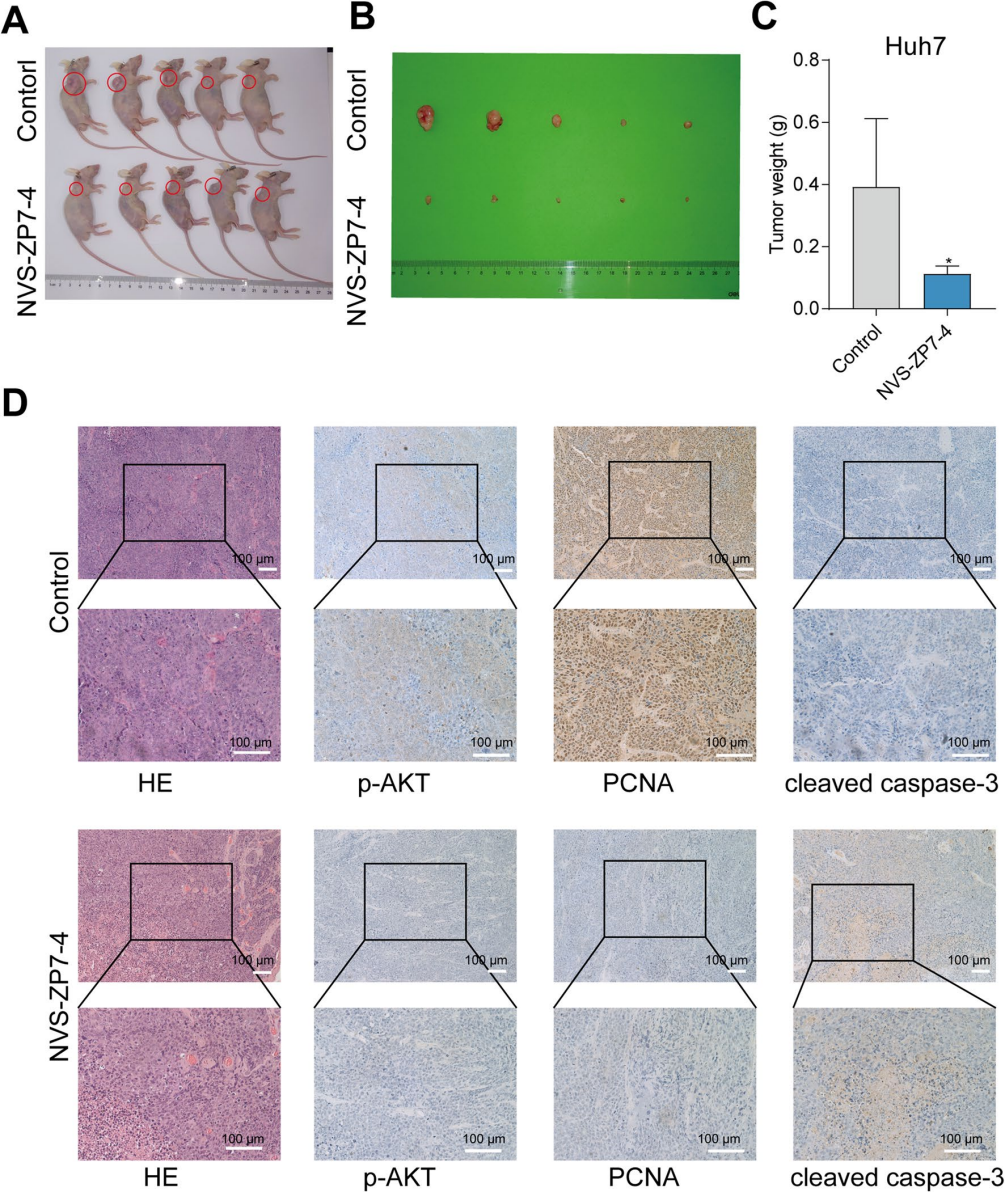
NVS-ZP7-4 inhibited HCC in vivo. (**A**,**B**) Subcutaneous tumor formation in Huh7 xenograft tumors in mice intraperitoneal injected with the NVS-ZP7-4 or DMSO for 2 weeks. (**C**) Quantification of the tumor weight was shown. (**D**) Representative HE staining, p-AKT and the proliferation marker PCNA, the apoptosis marker cleaved caspase-3 of xenografts treated with the NVS-ZP7-4 and DMSO. Scale bars: 100 μm. *p < 0.05 versus Control.

PI3K/AKT signaling has been identified to play a central role in tumor cell proliferation and cellular viability; thus, to elucidate the underlying mechanisms of the anti-tumor effect of NVS-ZP7-4, we further investigated the effects of NVS-ZP7-4 on the PI3K/ AKT pathway in HCCLM3 and Huh7 cells through western blotting. HCCLM3 and Huh7 cells were treated with DMSO, 1 μM NVS-ZP7-4, PI3K activator 740 Y-P, or a combination of 1 μM NVS-ZP7-4 and 740 Y-P. As shown in Fig. 6A, B, our results indicated that NVS-ZP7-4 could significantly suppress expression of p-AKT and p-ZIP7 in HCCLM3 and Huh7 cells; however, the expression of AKT and ZIP7 did not show a significant change. In addition, PI3K activator was used to evaluate the regulatory effect of NVS-ZP7-4 on the PI3K/AKT pathway. The blocking of NVS-ZP7-4 on PI3K/AKT was attenuated by PI3K activator, showing an elevated expression of p-AKT. Besides, Huh7 cells were used for further experiments. As shown in Fig. 6, NVS-ZP7-4 could significantly induce Huh7 cell apoptosis, decrease proportion of cells in G2, S phase, and repress cell proliferation, and those regulation was attenuated by PI3K activator, showing a decreased apoptosis, increased cell proliferation and proportion of cells in G2, S phase. Moreover, NVS-ZP7-4 treatment in vivo inhibited the expression of p-AKT in a xenograft nude mouse model (Fig. 7D). In conclusion, these results suggest that NVS-ZP7-4 significantly inhibits the activation of PI3K/AKT signaling and regulates cell fates (Supplementary Figures).

### NVS-ZP7-4 inhibited HCC in vivo

BALB/c nude mice were established to mimic the therapeutic efficacy of NVS-ZP7-4 in HCC. The in vivo results showed that NVS-ZP7-4 treatment significantly inhibited Huh7 tumor weight (Fig. 7A–C). The expression of PCNA is strongly related to DNA synthesis as a cofactor for DNA polymerase δ, thus remarkably correlating with the status of cellular proliferation. In our study, decreased PCNA expression in xenograft tumor tissues was also detected in the xenograft model treated with NVS-ZP7-4, suggesting that NVS-ZP7-4 significantly inhibited proliferation in vivo (Fig. 7D). These results implied that NVS-ZP7-4 is a potential therapeutic agent for the treatment of HCC.

## Discussion

HCC is the fastest growing cause of cancer-related death in the USA, with the highest mortality and incidence in East Asia and Africa; thus, it is crucial to develop a valid treatment strategy for HCC^10,11^. NVS-ZP7-4 was first discovered to inhibit ZIP7 in T cell acute lymphoblastic leukemia and was further investigated as a novel treatment for malignant tumors^3^. Our data demonstrated that NVS-ZP7-4 inhibits HCC growth both in vitro and in vivo, and may be a promising therapeutic target for HCC. Continuous proliferation, permanent replication, and activated invasion and metastasis are essential hallmarks of cancer, which equip cells with malignancy^33^. HCC also shares the vital characteristics of rapid proliferation and a high degree of cancer-related metastasis^10^. In our study, we revealed that NVS-ZP7-4 inhibited cellular proliferation, invasion, and migration of Huh7 and HCCLM3 cells, which supports the therapeutic efficiency of NVS-ZP7-4. We also showed that NVS-ZP7-4 causes G1 phase arrest and induces apoptosis, which might contribute to its growth inhibition.

Cancer resistance to apoptosis is essential in tumorigenesis; thus, targeting the apoptosis process might provide several novel therapies in oncology^33^. For example, the combination of X-linked inhibitor of apoptosis antisense oligonucleotide (AEG35156) with the first-line targeted drug sorafenib resulted in a moderate increase in progression-free survival and overall survival compared with sorafenib treatment alone^34^. However, although the mechanisms of both intrinsic and extrinsic pathways of apoptosis have been elaborated recently, it is still difficult to identify and select core apoptotic proteins for efficient targeted therapies^35,36^; thus, novel treatments must be investigated for further preclinical and clinical screening. In our study, we confirmed the induction of apoptosis after NVS-ZP7-4 treatment, which was consistent with a previous study in human colorectal cancer cells with knockdown of the targeted protein ZIP7^19,20^. The enhanced apoptosis due to NVS-ZP7-4 might be related to the elevated ER stress, since studies in multiple diseases also identified triggered ER stress after ZIP7 removal via genetic knockdown^37–39^, which finally overloaded the ER, triggering apoptosis^38^.

The PI3K/AKT signaling network participates in numerous feedback loops, counteracts various signaling, and provides ample opportunities to circumvent the effects of chemotherapy. Thus, combining drugs targeting the PI3K/AKT pathway is important for addressing chemotherapeutic resistance and establishing novel tumor treatments^40^. Significantly, activation of PI3K/AKT signaling was proven to be a major event in hepatocarcinogenesis^41–43^. In our study, we confirmed that NVS-ZP7-4 treatment functioned through inhibition of the PI3K/AKT pathway. Considering the important role of zinc as a second messenger, the inhibition of PI3K/AKT signaling might be attributed to the decreased zinc in the cytoplasm after NVS-ZP7-4 usage^31^. Similarly, overexpression of the NVS-ZP7-4 targeted protein ZIP7 caused activation of the AKT signaling pathway in gastric and breast cancer^31,44,45^.

Small molecules targeting the PI3K/AKT pathway, including PI3K/mTOR, pan-PI3K, and isoform-selective PI3K inhibitors, have been undergoing a large number of preclinical and clinical trials to investigate their efficacy and safety^40^. For example, a PI3K inhibitor suppressed cell proliferation and induced apoptosis in anlotinib-resistant osteosarcoma (OS) models and supports the PI3K inhibitor clinically used in anlotinib-refractory OS management^46^. Although multiple previous clinical trials did not provide promising evidence for clinical application in HCC and only some inhibitors of PI3K are currently approved for the clinical treatment of human cancers^42,47^, the PI3K/AKT pathway still renders attractive therapeutic options for HCC treatments. Thus, testing new agents based on basic science and clinical trials would provide more opportunities for further research in HCC.

The treatment of HCC is decided according to the tumor stage as per the Barcelona Clinic Liver Cancer staging system^48^. However, with most HCC patients diagnosed in the advanced stage of the disease, treatment options are limited and lead to poor prognosis. Chemotherapy is an important tool, but it also has a high rate of drug resistance^49^. The only approved first-line therapy with sorafenib has shown limited survival benefits and poor tolerability^10^. Notably, our in vivo experiments identified that NVS-ZP7-4 treatment of HCC in nude mice decreased tumor weight, further implying its therapeutic potential by repressing the PI3K/AKT pathway in HCC. To the best of our knowledge, this is the first study to confirm the therapeutic role of NVS-ZP7-4 in HCC and identify that NVS-ZP7-4 acts as an inhibitor targeting PI3K/AKT. Therefore, our study provides potential prospects for independent or combined treatment of HCC with NVS-ZP7-4. However, we noticed that there are some limitations in our study. Firstly, we have not proved the direct mechanisms underlying NVS-ZP7-4, so more experiments are needed in the future to verify its effectiveness. Secondly, we should explore the heterogeneity of HCC treatment of NVS-ZP7-4 based on different cell lines and provide more information for clinical application.

## Conclusion

In conclusion, our study verified the effects of NVS-ZP7-4 on HCC cell proliferation, migration, invasion, apoptosis, and cell cycle progression. Furthermore, NVS-ZP7-4 inhibited the activation of PI3K/AKT signaling, and in vivo, NVS-ZP7-4 treatment of HCC in nude mice resulted in decreased tumor weight and expression of PCAN, p-AKT, and increased cleaved caspase-3. Thus, our study provides a novel treatment for HCC in addition to targeted therapy, chemotherapy, and other treatment strategies and provides a research basis for elucidating the underlying mechanisms of NVS-ZP7-4 in HCC.

## Supplementary Information

Supplementary Figures.

## Data Availability

This manuscript has not been published previously and is not under consideration by another journal. The authors declare that they have no competing interests. The data generated or analyzed during this study are available from the corresponding author on reasonable request.
