# Supplementary material for: NVS-ZP7-4 inhibits hepatocellular carcinoma tumorigenesis and promotes apoptosis via PI3K/AKT signaling
Source: Sci Rep. 2023 Jul 21;13:11795. doi: 10.1038/s41598-023-38596-7 (PMC10362011; doi:10.1038/s41598-023-38596-7)
Supplement: Supplementary file 1 — Supplementary Figures. [file 41598_2023_38596_MOESM1_ESM.pdf]

Supplementary Figures

NVS-ZP7-4 Inhibits Hepatocellular Carcinoma Tumorigenesis and Promotes Apoptosis via PI3K/AKT Signaling

Qing Tong[1†], Dong Yan[1†], Yan Cao[1], Xiaogang Dong[1], Yimamumaimaitijiang Abula[1], Huan Yang[1], Panpan Kong[1] and Mingyu Yi[1,2‡]

[1] Department of Hepatopancreatobiliary Surgery, Affiliated Cancer Hospital of Xinjiang Medical University, Urumqi, Xinjiang, China

[2] Department of Anesthesiology, The Third Xiangya Hospital of Central South University, Hunan, China.

‡ **Correspondence:**
Mingyu Yi
Address: Third Xiangya Hospital of Central South University, No. 138, tongzipo Road, Changsha City, Hunan Province, China, 410000
Email: ymy0408@csu.edu.cn

†These authors have contributed equally to this work and share first authorship.

Figure 3

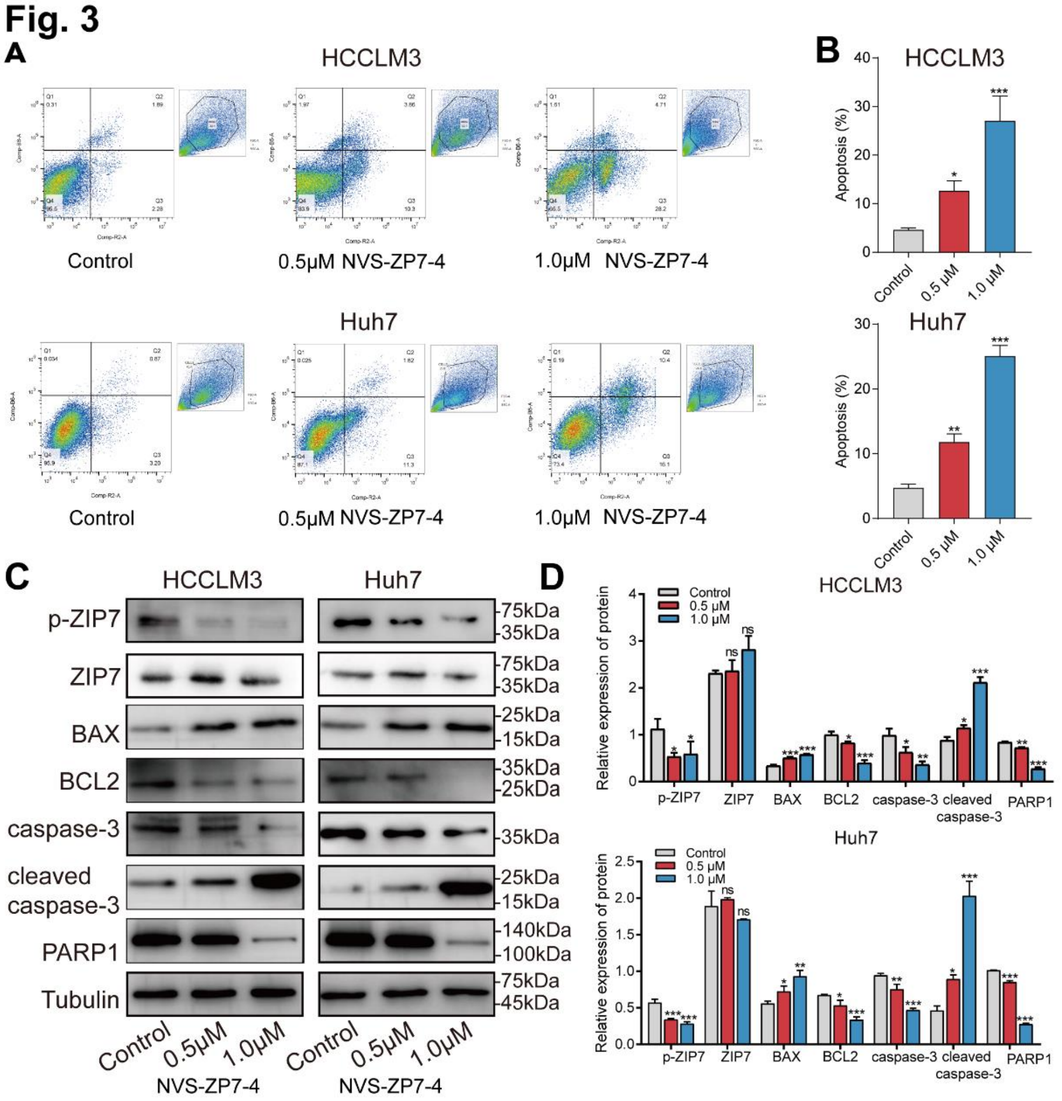

Fig. 3
A
HCCLM3
Control
0.5μM NVS-ZP7-4
1.0μM NVS-ZP7-4
Huh7
Control
0.5μM NVS-ZP7-4
1.0μM NVS-ZP7-4
B
HCCLM3
Apoptosis (%)
Control
0.5 μM
1.0 μM
Huh7
Apoptosis (%)
Control
0.5 μM
1.0 μM
C
HCCLM3
Huh7
p-ZIP7
ZIP7
BAX
BCL2
caspase-3
cleaved caspase-3
PARP1
Tubulin
75kDa
35kDa
75kDa
35kDa
25kDa
15kDa
35kDa
25kDa
35kDa
25kDa
15kDa
140kDa
100kDa
75kDa
45kDa
Control
0.5μM
1.0μM
NVS-ZP7-4
Control
0.5μM
1.0μM
NVS-ZP7-4
D
HCCLM3
Relative expression of protein
Control
0.5 μM
1.0 μM
p-ZIP7
ZIP7
BAX
BCL2
caspase-3
cleaved caspase-3
PARP1
Huh7
Relative expression of protein
Control
0.5 μM
1.0 μM
p-ZIP7
ZIP7
BAX
BCL2
caspase-3
cleaved caspase-3
PARP1

Figure S1

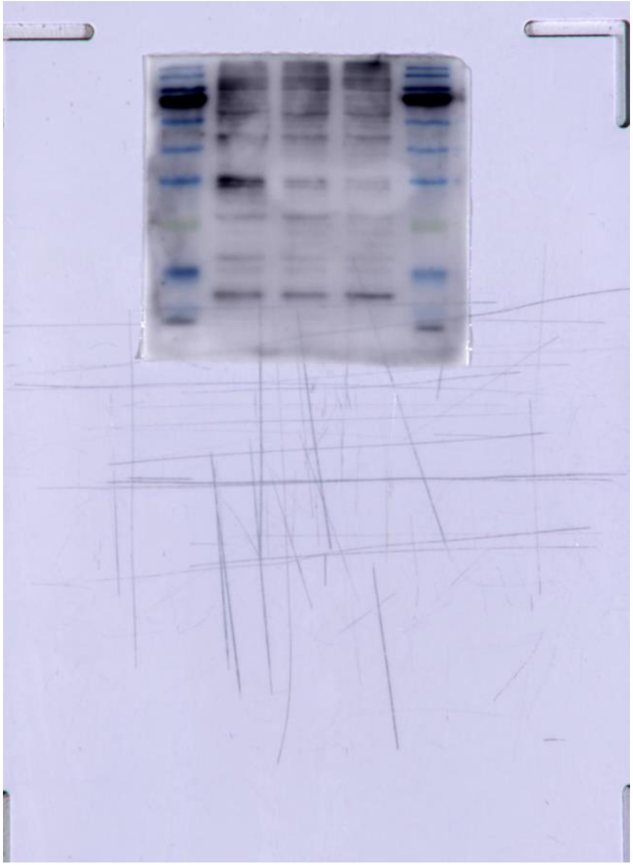

Figure S1 shows the gel and blotting of p-ZIP7 in HCCLM3 cells in each group, from left to right are groups Control, NVS-ZP7-4 0.5 µM, NVS-ZP7-4 1.0 µM. (gels/blots of p-ZIP7 in Figure 3)

Figure S2

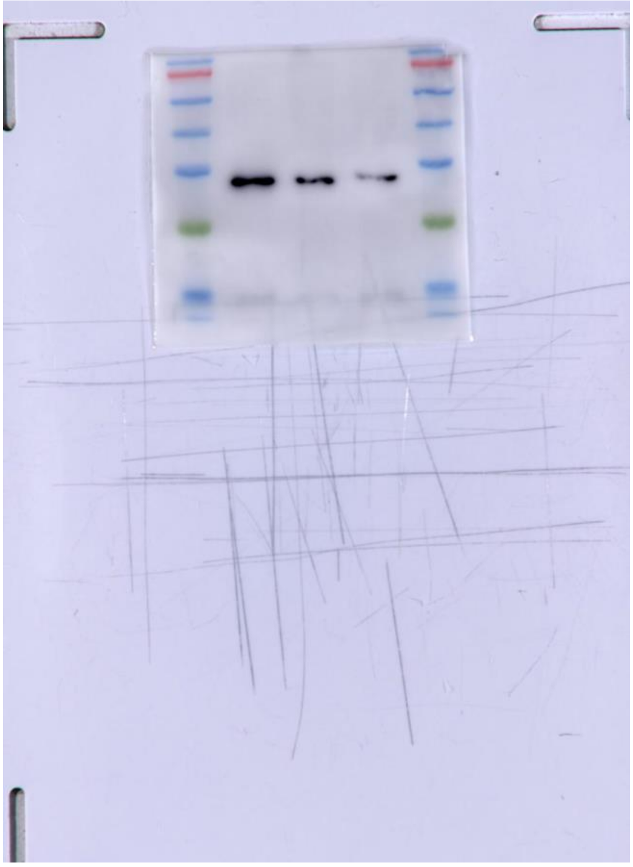

Figure S2 shows the gel and blotting of p-ZIP7 in Huh7 cells in each group, from left to right are groups Control, NVS-ZP7-4 0.5 μM, NVS-ZP7-4 1.0 μM. (gels/blots of p-ZIP7 in Figure 3)

Figure S3

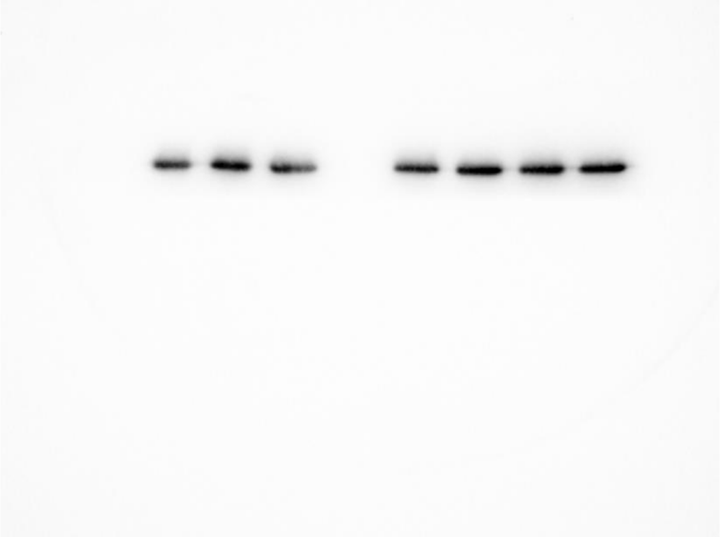

Figure S3 shows the gel and blotting of ZIP7 in HCCLM3 cells in each group, from left to right are groups Control, NVS-ZP7-4 0.5 µM, NVS-ZP7-4 1.0 µM, and groups Control, 740 Y-P, NVS-ZP7-4, Combination. (gels/blots of ZIP7 in Figure 3 and Figure 6)

Figure S4

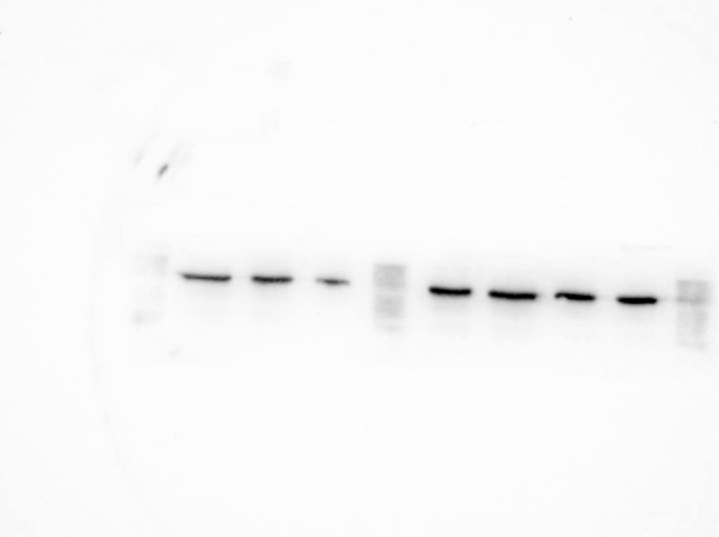

Figure S4 shows the gel and blotting of ZIP7 in Huh7 cells in each group, from left to right are groups Control, NVS-ZP7-4 0.5 µM, NVS-ZP7-4 1.0 µM, and groups Control, 740 Y-P, NVS-ZP7-4, Combination. (gels/blots of ZIP7 in Figure 3 and Figure 6)

Figure S5

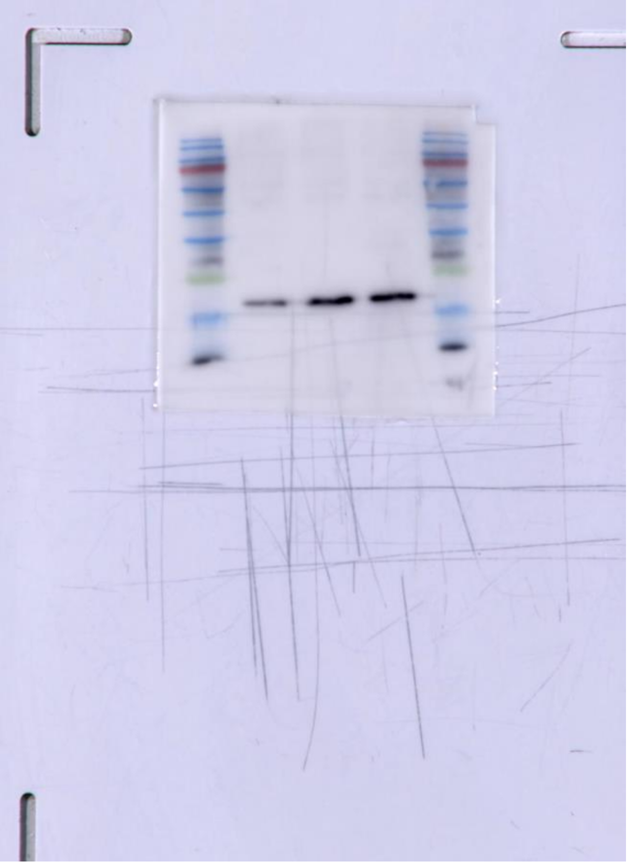

Figure S5 shows the gel and blotting of BAX in HCCLM3 cells in each group, from left to right are groups Control, NVS-ZP7-4 0.5 μM, NVS-ZP7-4 1.0 μM. (gels/blots of BAX in Figure 3)

Figure S6

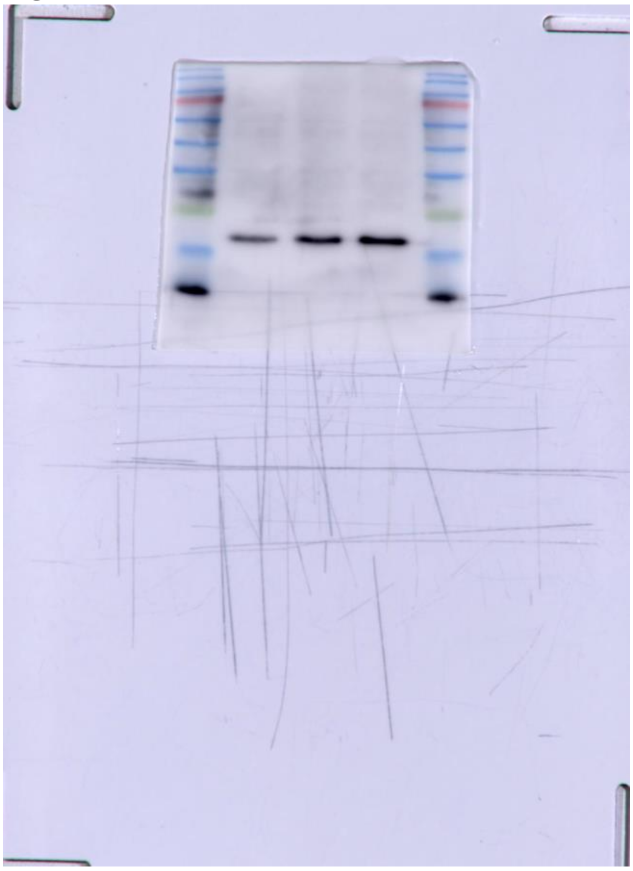

Figure S6 shows the gel and blotting of BAX in Huh7 cells in each group, from left to right are groups Control, NVS-ZP7-4 0.5 μM, NVS-ZP7-4 1.0 μM. (gels/blots of BAX in Figure 3)

Figure S7

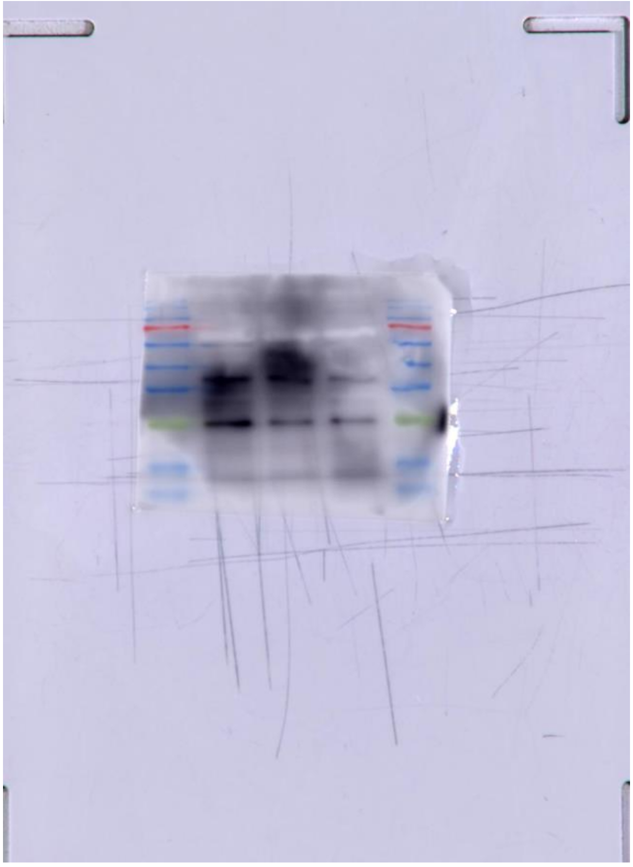

Figure S7 shows the gel and blotting of BCL2 in HCCLM3 cells in each group, from left to right are groups Control, NVS-ZP7-4 0.5 μM, NVS-ZP7-4 1.0 μM. (gels/blots of BCL2 in Figure 3)

Figure S8

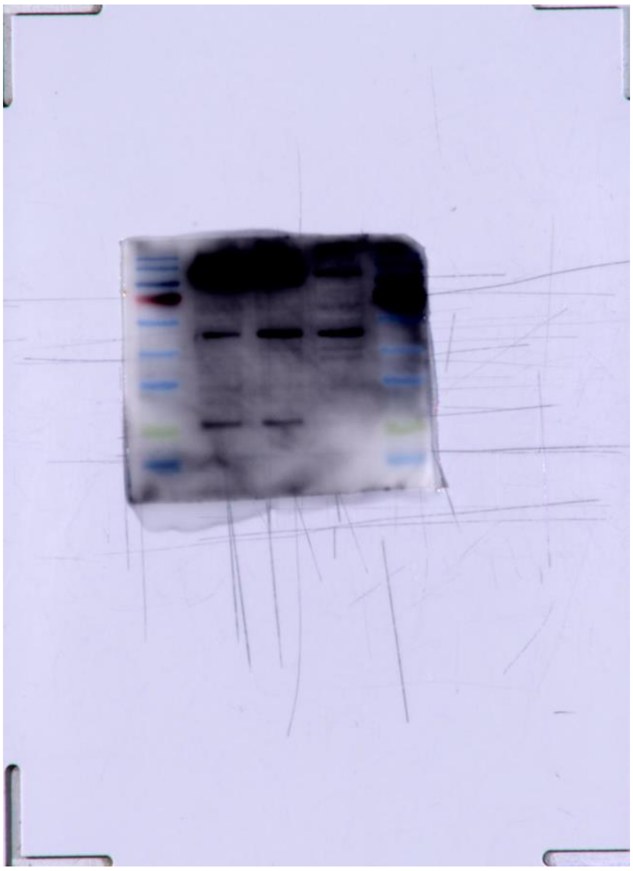

Figure S8 shows the gel and blotting of BCL2 in Huh7 cells in each group, from left to right are groups Control, NVS-ZP7-4 0.5 μM, NVS-ZP7-4 1.0 μM. (gels/blots of BCL2 in Figure 3)

Figure S9

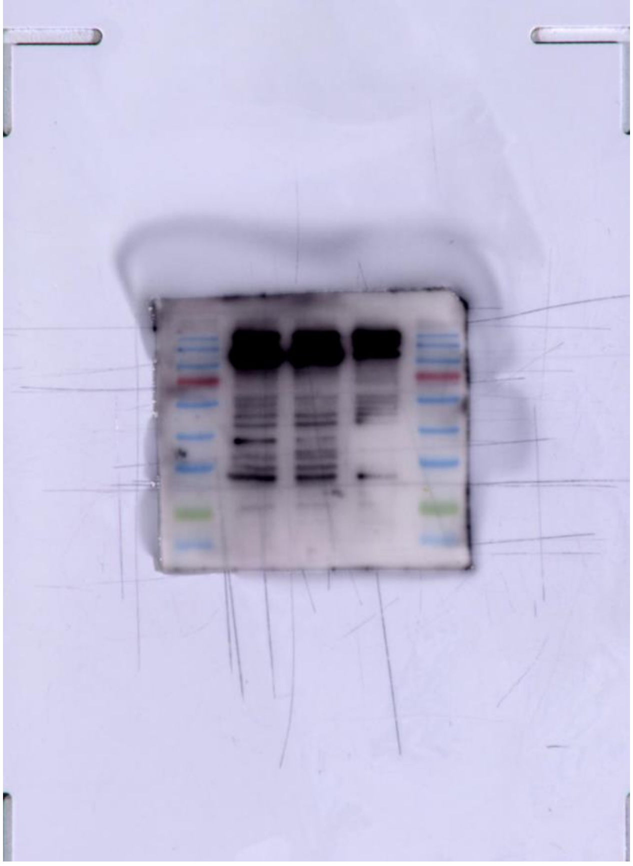

Figure S9 shows the gel and blotting of caspase-3 in HCCLM3 cells in each group, from left to right are groups Control, NVS-ZP7-4 0.5 μM, NVS-ZP7-4 1.0 μM. (gels/blots of caspase-3 in Figure 3)

Figure S10

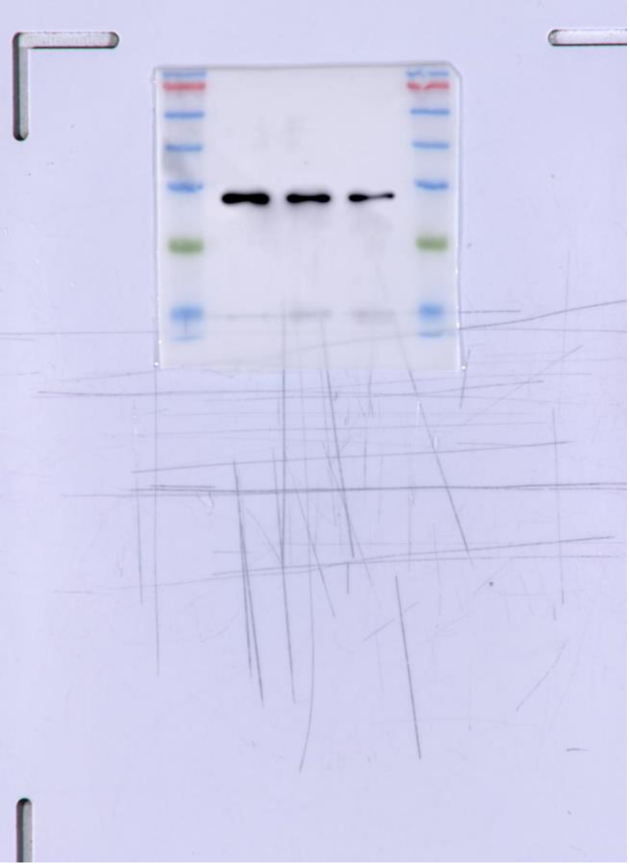

Figure S10 shows the gel and blotting of caspase-3 in Huh7 cells in each group, from left to right are groups Control, NVS-ZP7-4 0.5 μM, NVS-ZP7-4 1.0 μM. (gels/blots of caspase-3 in Figure 3)

Figure S11

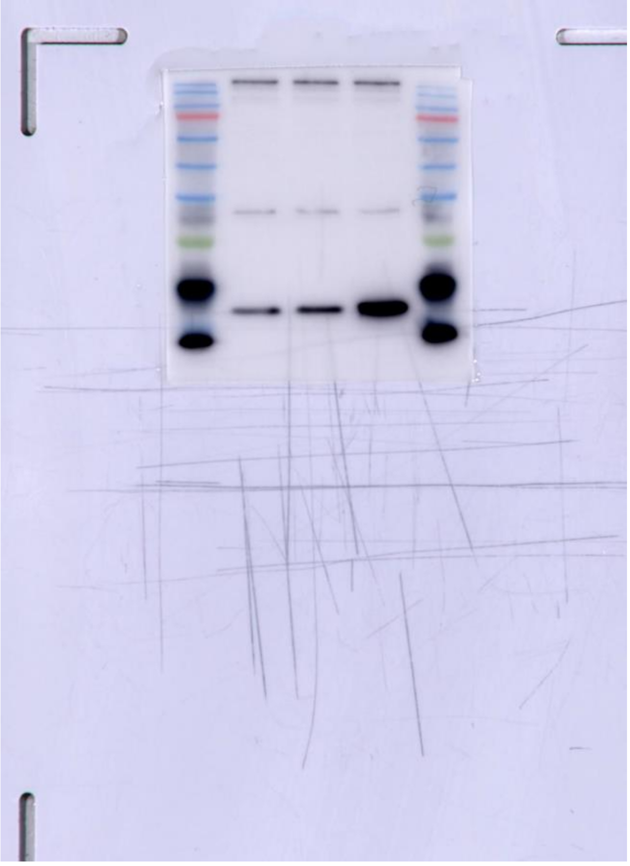

Figure S11 shows the gel and blotting of cleaved caspase-3 in HCCLM3 cells in each group, from left to right are groups Control, NVS-ZP7-4 0.5 μM, NVS-ZP7-4 1.0 μM. (gels/blots of cleaved caspase-3 in Figure 3)

Figure S12

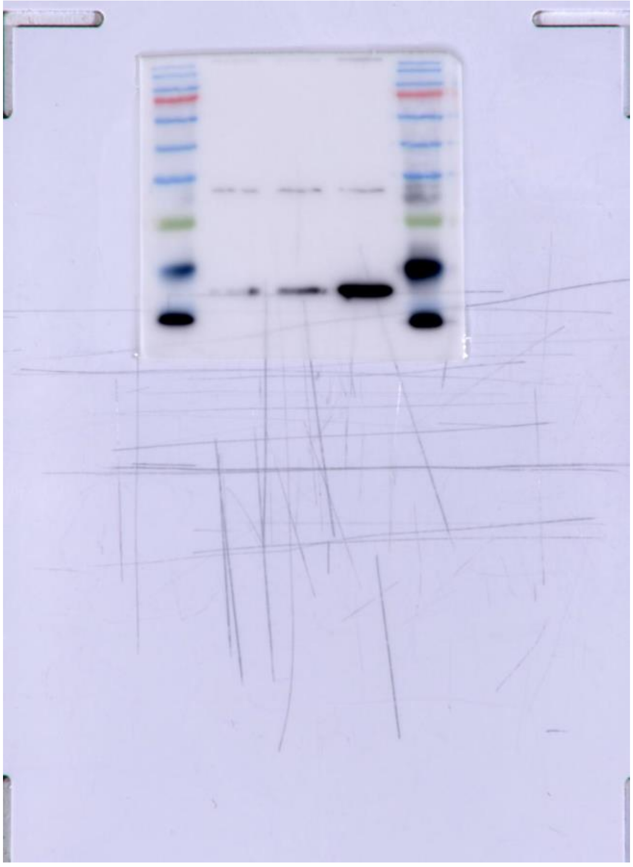

Figure S12 shows the gel and blotting of cleaved caspase-3 in Huh7 cells in each group, from left to right are groups Control, NVS-ZP7-4 0.5 µM, NVS-ZP7-4 1.0 µM. (gels/blots of cleaved caspase-3 in Figure 3)

Figure S13

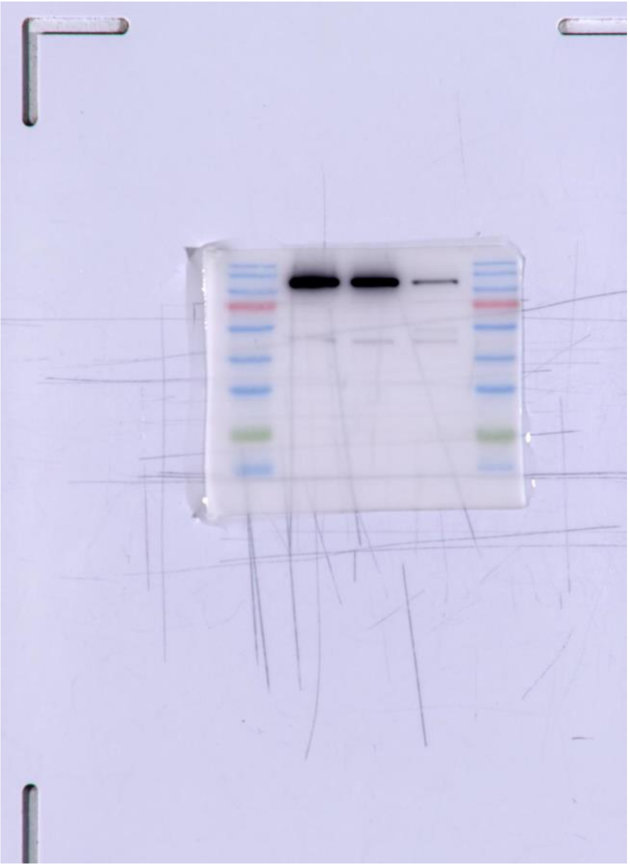

Figure S13 shows the gel and blotting of PARP1 in HCCLM3 cells in each group, from left to right are groups Control, NVS-ZP7-4 0.5 μM, NVS-ZP7-4 1.0 μM. (gels/blots of PARP1 in Figure 3)

Figure S14

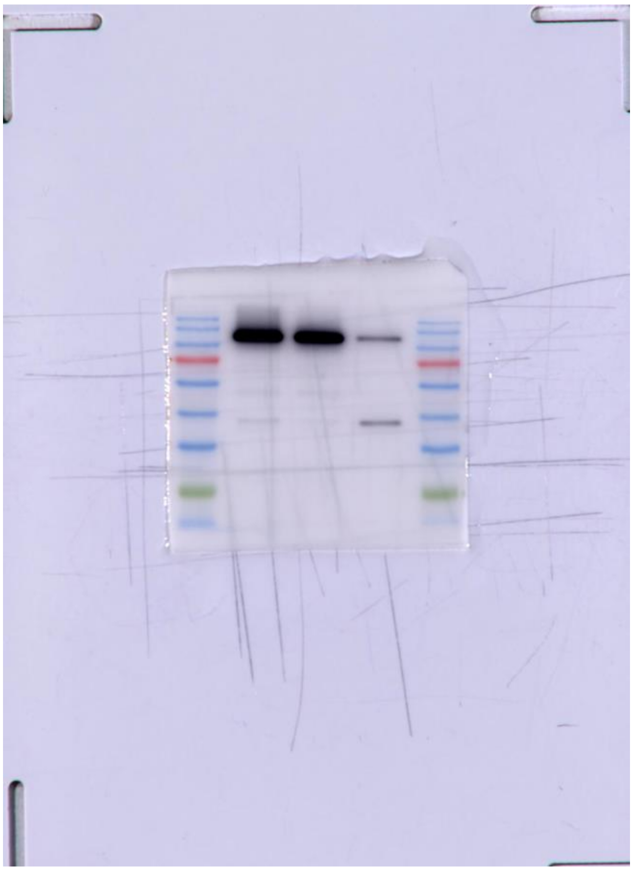

Figure S14 shows the gel and blotting of PARP1 in Huh7 cells in each group, from left to right are groups Control, NVS-ZP7-4 0.5 μM, NVS-ZP7-4 1.0 μM. (gels/blots of PARP1 in Figure 3)

Figure S15

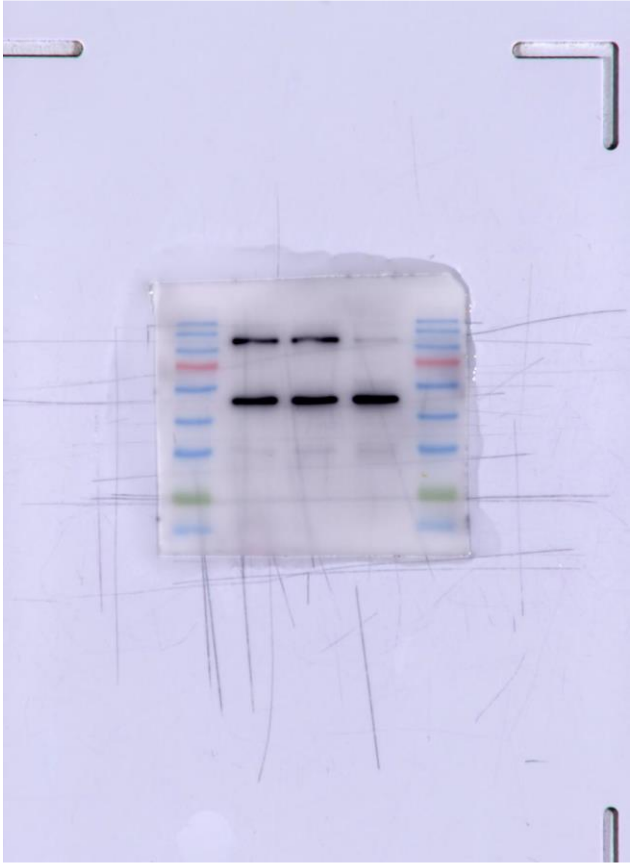

Figure S15 shows the gel and blotting of Tubulin in HCCLM3 cells in each group, from left to right are groups Control, NVS-ZP7-4 0.5 µM, NVS-ZP7-4 1.0 µM. (gels/blots of Tubulin in Figure 3)

Figure S16

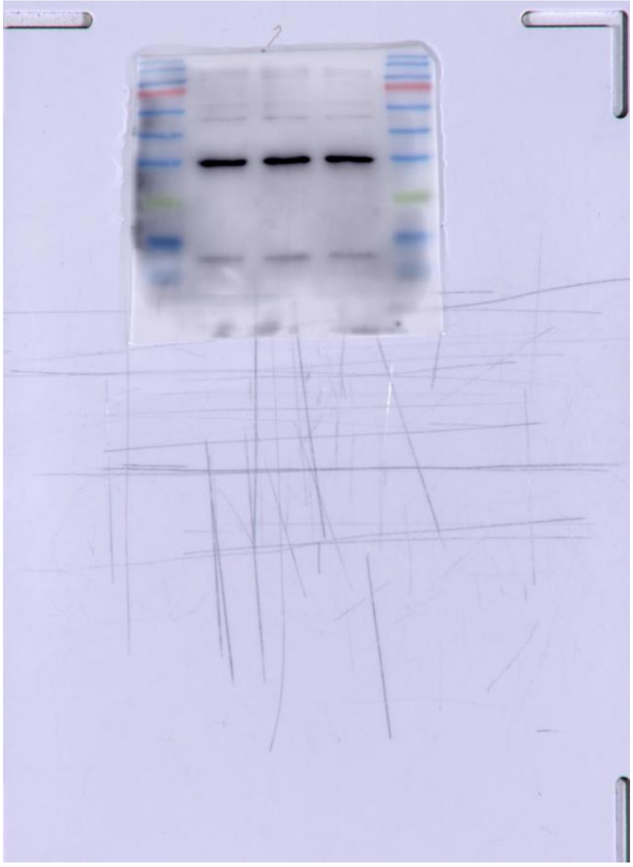

Figure S16 shows the gel and blotting of Tubulin in Huh7 cells in each group, from left to right are groups Control, NVS-ZP7-4 0.5 μM, NVS-ZP7-4 1.0 μM. (gels/blots of Tubulin in Figure 3)

Figure 6

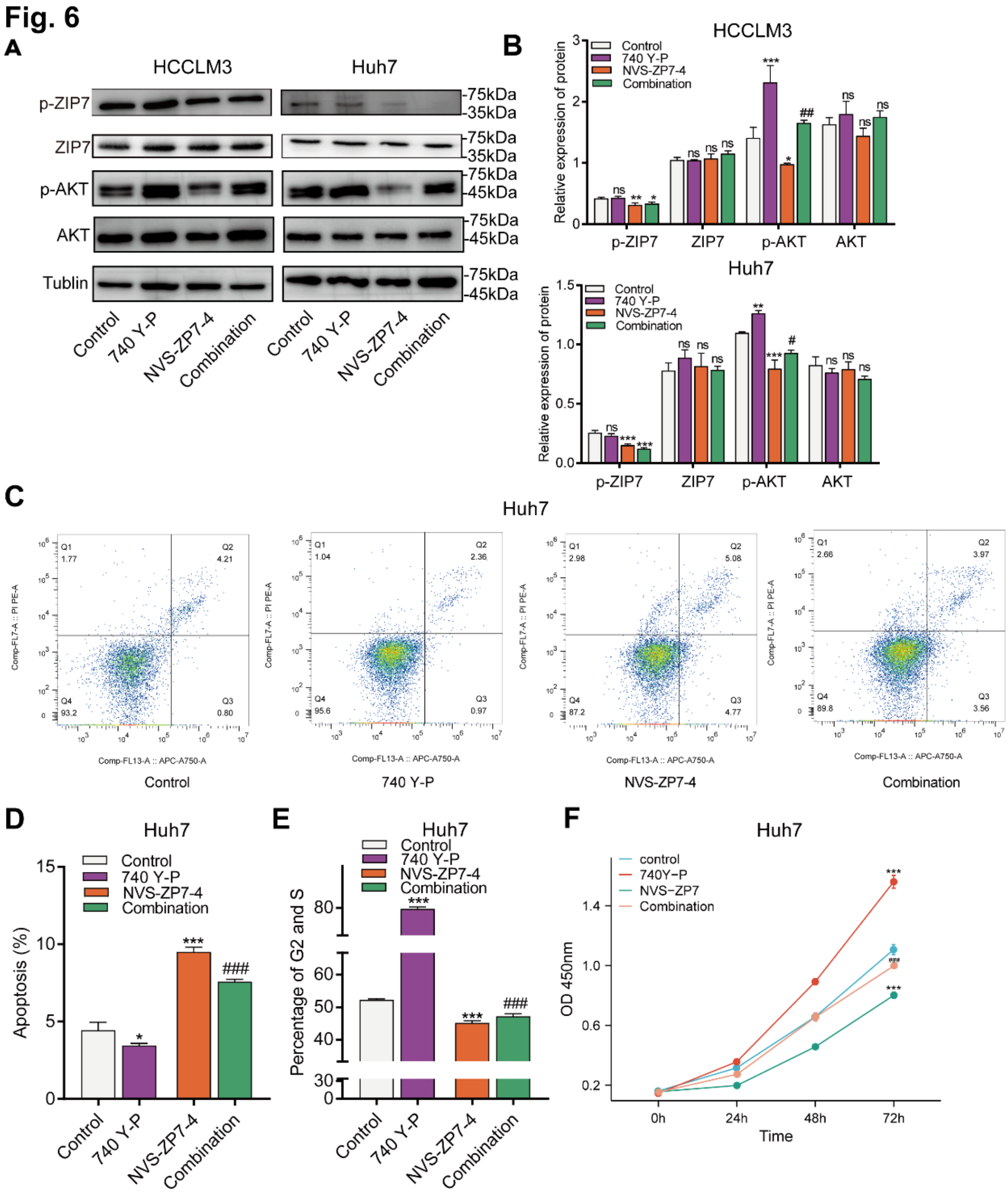

Fig. 6
A
HCCLM3
Huh7
p-ZIP7
ZIP7
p-AKT
AKT
Tublin
-75kDa
-35kDa
-45kDa
Control
740 Y-P
NVS-ZP7-4
Combination
B
HCCLM3
Huh7
Relative expression of protein
p-ZIP7
ZIP7
p-AKT
AKT
C
Huh7
Comp-FL7-A :: PI PE-A
Comp-FL13-A :: APC-A750-A
Q1 1.77
Q2 4.21
Q3 0.80
Q4 93.2
Q1 1.04
Q2 2.36
Q3 0.97
Q4 95.6
Q1 2.98
Q2 5.08
Q3 4.77
Q4 87.2
Q1 2.66
Q2 3.97
Q3 3.56
Q4 89.8
Control
740 Y-P
NVS-ZP7-4
Combination
D
Huh7
Apoptosis (%)
E
Huh7
Percentage of G2 and S
F
Huh7
control
740Y-P
NVS-ZP7
Combination
OD 450nm
Time
0h
24h
48h
72h

Figure S17

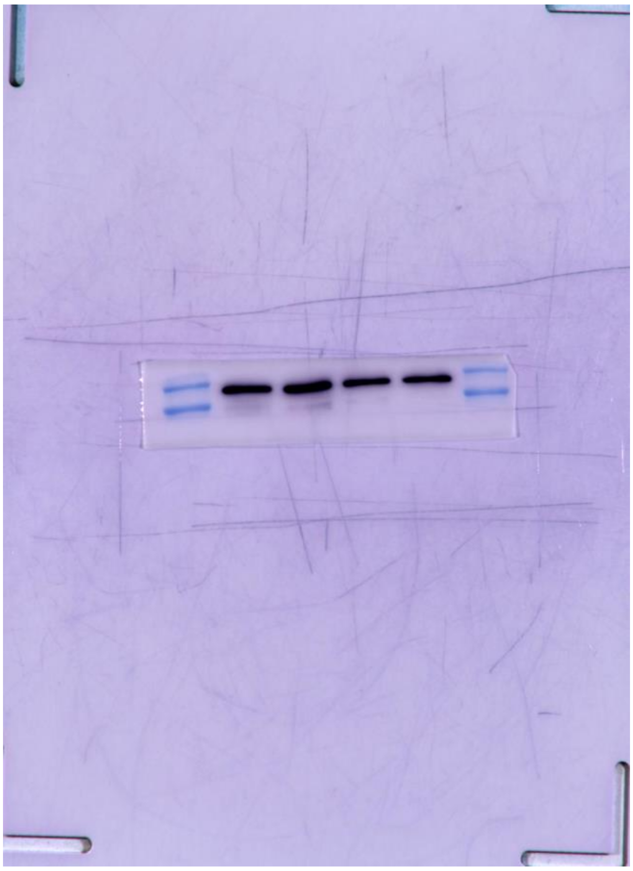

Figure S17 shows the gel and blotting of p-ZIP7 in HCCLM3 cells in each group, from left to right are groups Control, 740 Y-P, NVS-ZP7-4, Combination. (gels/blots of p-ZIP7 in Figure 6)

Figure S18

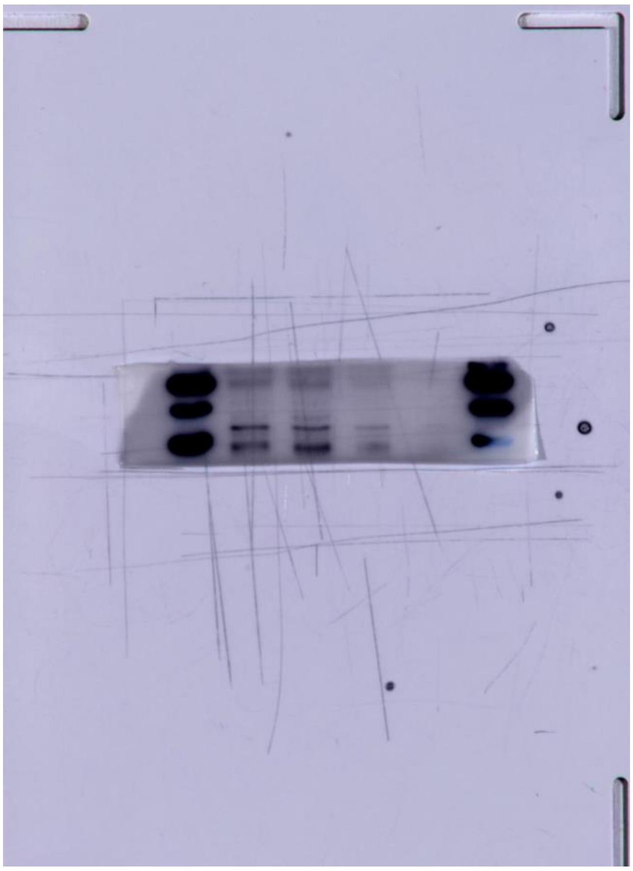

Figure S18 shows the gel and blotting of p-ZIP7 in Huh7 cells in each group, from left to right are groups Control, 740 Y-P, NVS-ZP7-4, Combination. (gels/blots of p-ZIP7 in Figure 6)

Figure S19

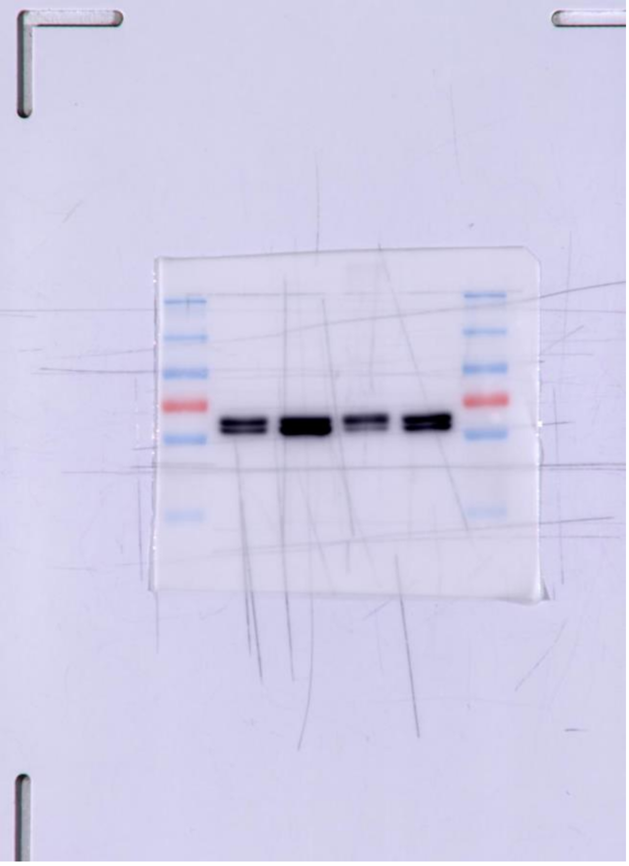

Figure S19 shows the gel and blotting of p-AKT in HCCLM3 cells in each group, from left to right are groups Control, 740 Y-P, NVS-ZP7-4, Combination. (gels/blots of p-AKT in Figure 6)

Figure S20

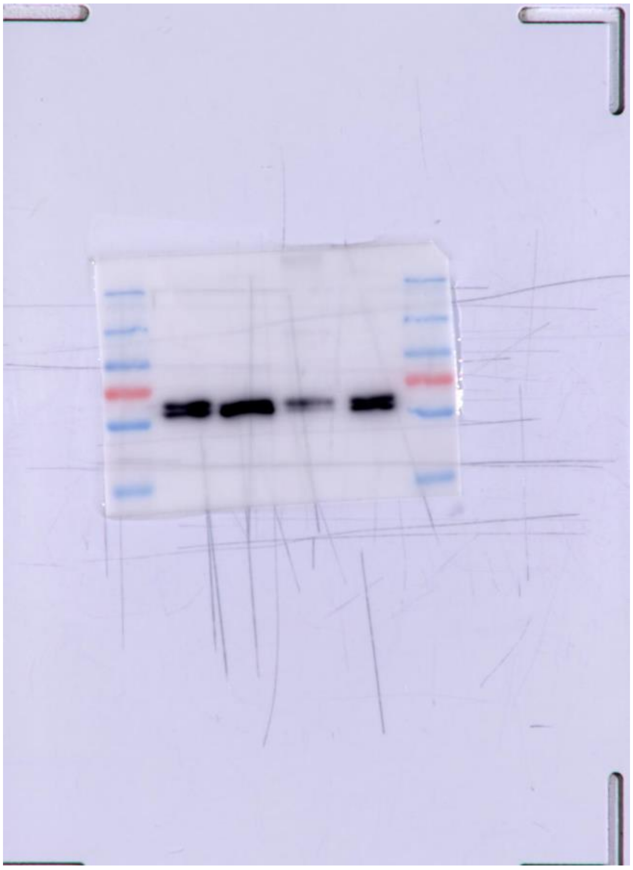

Figure S20 shows the gel and blotting of p-AKT in Huh7 cells in each group, from left to right are groups Control, 740 Y-P, NVS-ZP7-4, Combination. (gels/blots of p-AKT in Figure 6)

Figure S21

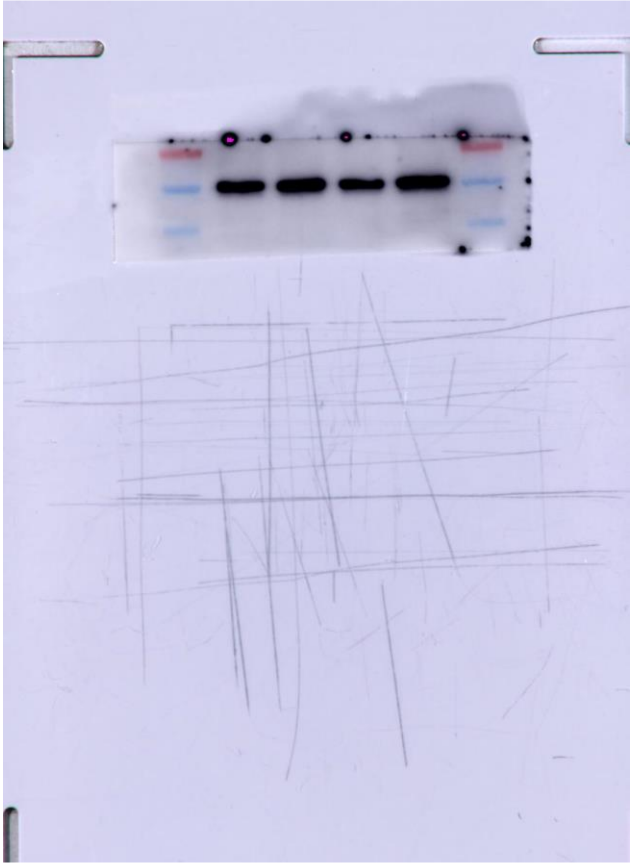

Figure S21 shows the gel and blotting of AKT in HCCLM3 cells in each group, from left to right are groups Control, 740 Y-P, NVS-ZP7-4, Combination. (gels/blots of AKT in Figure 6)

Figure S22

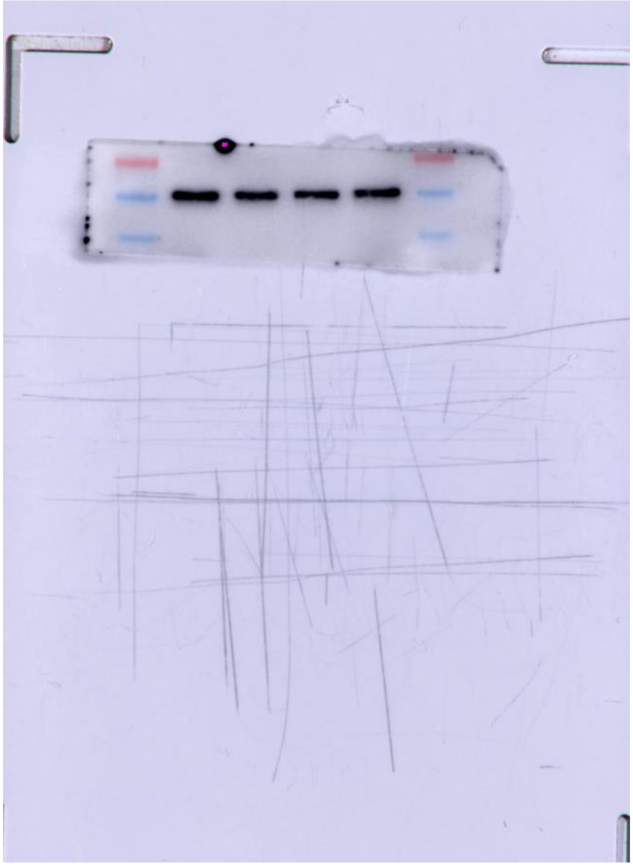

Figure S22 shows the gel and blotting of AKT in Huh7 cells in each group, from left to right are groups Control, 740 Y-P, NVS-ZP7-4, Combination. (gels/blots of AKT in Figure 6)

Figure S23

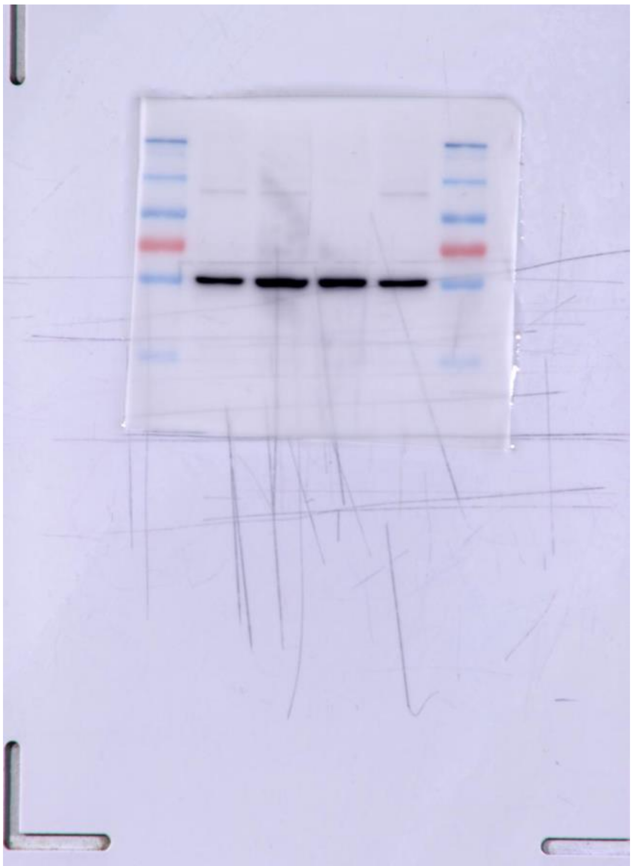

Figure S23 shows the gel and blotting of Tubulin in HCCLM3 cells in each group, from left to right are groups Control, 740 Y-P, NVS-ZP7-4, Combination. (gels/blots of Tubulin in Figure 6)

Figure S24

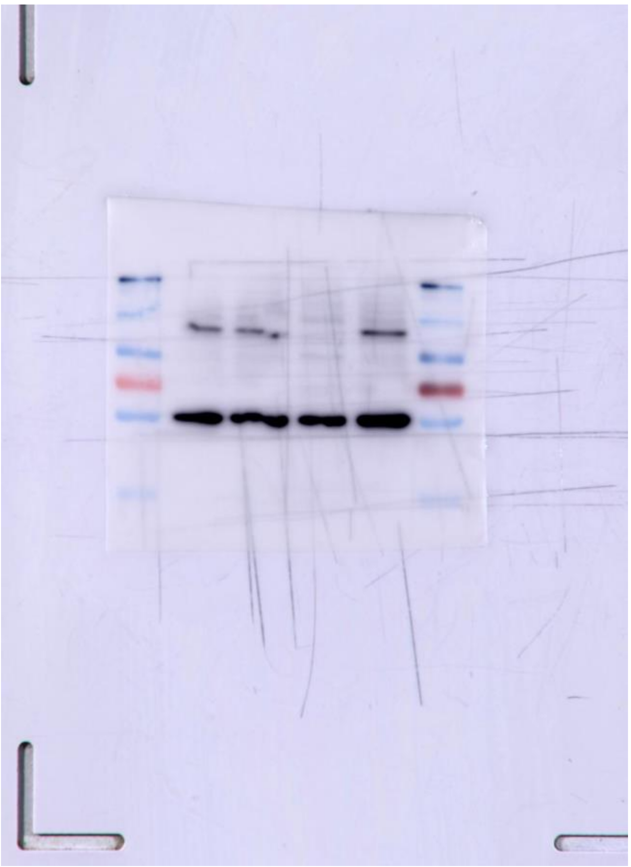

Figure S24 shows the gel and blotting of Tubulin in Huh7 cells in each group, from left to right are groups Control, 740 Y-P, NVS-ZP7-4, Combination. (gels/blots of Tubulin in Figure 6)
